# Histone deacetylase 9 deficiency exaggerates uterine M2 macrophage polarization

**DOI:** 10.1111/jcmm.16616

**Published:** 2021-06-19

**Authors:** Yanqin Liu, Meirong Du, Hai‐Yan Lin

**Affiliations:** ^1^ State Key Laboratory of Stem Cell and Reproductive Biology Institute of Zoology Chinese Academy of Sciences Beijing China; ^2^ Institute of Physical Science and Information Technology Anhui University Hefei China; ^3^ State Key Laboratory of Medicinal Chemical Biology and College of Life Sciences Nankai University Tianjin China; ^4^ Gynecology and Obstetrics Hospital Fudan University Shanghai China; ^5^ Key Laboratory of Zoological Systematics and Evolution Institute of Zoology Chinese Academy of Sciences Beijing China; ^6^ Present address: National Laboratory of Biomacromolecules CAS Center for Excellence in Biomacromolecules Institute of Biophysics Chinese Academy of Sciences Beijing China

**Keywords:** abortion, histone deacetylase 9, macrophage polarization, pregnancy, uterine macrophage

## Abstract

The maternal‐foetal interface is an immune‐privileged site where the semi‐allogeneic embryo is protected from attacks by the maternal immune system. Uterine macrophages are key players in establishing and maintaining pregnancy, and the dysregulation of the M1‐M2 subpopulation balance causes abortion. We separated two distinct mouse uterine macrophage subpopulations during early pregnancy, CD45^+^F4/80^+^CD206^−^ M1‐like (M1) and CD45^+^F4/80^+^CD206^+^ M2‐like (M2) cells. The M1 preponderance was significantly exaggerated at 6 hours after lipopolysaccharide (LPS) treatment, and adoptive transfer of M2 macrophages partially rescued LPS‐induced abortion. RNA sequencing analysis of mouse uterine M2 versus M1 revealed 1837 differentially expressed genes (DEGs), among which 629 was up‐regulated and 1208 was down‐regulated. Histone deacetylase 9 (*Hdac9*) was one of the DEGs and validated to be significantly up‐regulated in uterine M2 as compared with M1. Remarkably, this differential expression profile between M1 and M2 was also evident in primary splenic macrophages and in vitro polarized murine peritoneal, bone marrow–derived and RAW 264.7 macrophages. In *Hdac9/HDAC9* knockout RAW 264.7 and human THP‐1–derived macrophages, the expression of M1 differentiation markers was unchanged or decreased whereas M2 markers were increased compared with the wild‐type cells, and these effects were unrelated to compromised proliferation. Furthermore, *Hdac9*/*HDAC9* ablation significantly enhanced the phagocytosis of fluorescent microspheres in M2 Raw 264.7 cells yet decreased the capacity of THP‐1‐derived M1 macrophages. The above results demonstrate that *Hdac9*/*HDAC9* deficiency exaggerates M2 macrophage polarization in mouse and human macrophages, which may provide clues for our understanding of the epigenetic regulation on macrophage M1/M2 polarization in maternal‐foetal tolerance.

## 1 INTRODUCTION

The maternal‐foetal interface is a privileged site for co‐ordinating the process of immune tolerance to protect the genetically foreign semi‐allogeneic embryo from attack by the maternal immune system. The immune cells that reside in the decidua, which surrounds the placenta and conceptus, are highly specialized and important for the establishment of immune microenvironment during pregnancy. Macrophages are the second most abundant immune cell population in the pregnant uterus (known as decidua), comprising approximately 20% of the total decidual leucocyte population.^1^, ^2^ Successful pregnancy requires maintenance of decidual macrophages (dMϕs) at homeostatic conditions, thus functioning as crucial regulators of maternal‐foetal tolerance.

Tissue macrophages not only play a central role in host defence, but also maintain homeostatic functions in tissue remodelling. Two widely appreciated states of macrophage polarization, classical M1 activation induced by lipopolysaccharide (LPS) and interferon gamma (IFNG) and alternative M2 activation stimulated by interleukin (IL)4 and/or IL13, represent two ends of a functional differentiation spectrum. In response to infectious pathogens, macrophages undergo M1 activation and induce Th1 immunity, secreting pro‐inflammatory cytokines, nitric oxide (NO), reactive oxygen species, proteolytic enzymes, etc On the other hand, macrophages also have the ability to control immune responses, producing anti‐inflammatory cytokines such as IL10 and factors promoting tissue remodelling.^2^, ^3^ Yet, it is also increasingly recognized that other polarization phenotypes of macrophages also exist in a tissue‐specific manner, due to their high diversity, plasticity and heterogeneity. For example, M2 macrophages can be subdivided into M2a, M2b, M2c and M2d, and functions of macrophages in inflammation versus immune regulation are not completely attributable to one or the other subset.^1^

Similarly, dMϕs are differentiated or polarized into two distinct subpopulations, M1 and M2.^4^ Appropriately and timely regulated M1/M2 polarization has been considered a key player in establishing and maintaining pregnancy during different phases of gestation, and the dysregulation of this balance is correlated with recurrent spontaneous abortion (RSA) in humans.^5^, ^6^ They are polarized towards the predominant M1 phenotype during the embryo implantation window, switch to a balanced M1/M2 profile during placenta development and uterine vasculature remodelling, and shift towards M2 polarization for pregnancy maintenance.^7^ Different dMϕ subsets with distinct functional properties have been identified by flow cytometric studies. In the first‐trimester human decidua, for example, dMϕs can be discriminated into CD14^+^ICAM‐3^+^ and CD14^+^ICAM‐3^−^ subsets. The CD14^+^ICAM‐3^−^ population expresses high levels of CD163, CD206, CD209 and NRP‐1 and low levels of CD11c, displaying more pronounced M2 phenotype. In contrast, the CD14^+^ICAM‐3^+^ population is CD163‐, CD206‐, CD209‐ and NRP‐1‐negative and expresses high levels of CD11c.^8^ Thus, expression of ICAM‐3 and CD11c correlates well with each other.^8^ Another report shows that two distinct subsets of CD14^+^CD11c^hi^ and CD14^+^CD11c^lo^ express genes associated with inflammation and extracellular matrix formation, respectively; however, these two subpopulations secret both pro‐inflammatory and anti‐inflammatory cytokines, therefore do not fit the conventional M1/M2 profile.^9^ With respect to mouse uterine macrophages, two abundant populations of F4/80^+^MHCII^hi^ and F4/80^+^MHCII^lo^ which differentially express M1 and M2 markers^10^ have been identified.

Given that dMϕs contribute to the balance that establishes maternal‐foetal tolerance, it is important to understand how their tolerogenic phenotypes are induced in a juxtacrine or paracrine manner. Human placenta–derived M‐CSF and IL10 can induce dMϕ to differentiate towards homeostatic CD14^+^CD163^+^CD206^+^CD209^+^ M2 phenotype, producing IL10 and CCL18.^11^ Soluble human leucocyte antigen G5 (sHLAG5) secreted by trophoblasts reduces the expression of M1 marker CD86 and increases the expression of M2 marker CD163 and the phagocytic activity.^12^ Human trophoblast and decidual stromal cells–secreted RANKL (nuclear factor‐κB ligand) polarizes dMϕ towards M2 phenotype via activating AKT/STAT6 (signal transducer and activator of transcription 6) signalling, and depletion of RANKL results in abnormal phenotypes of dMϕ in vivo and increased foetal loss rates in mice.^13^ In light of these important findings, the mechanisms of dMϕ differentiation in the local homeostatic microenvironment at the maternal‐foetal interface remain to be further explored.

A large body of emerging evidence suggests that epigenetic regulation is of crucial importance for conversion of polarization signalling pathways into complex and sustained gene expression patterns in macrophages and for determination of functional outcome in response to environmental stimulation based on previous transcriptional memory. Together with transcriptional regulation, epigenetic regulation of inflammatory cytokine gene loci in macrophages is present at three states: repressed, poised and activated states, which are characterized by a closed chromatin structure occupied by negative histone marks, active histone marks facilitating partially open chromatin configuration and active histone marks plus an open chromatin conformation, respectively.^14^ Among these epigenetic modifications, histone acetylation is mediated by histone acetyltransferases, which use acetyl‐CoA to modify ε‐amino group of lysine residues on histone proteins, resulting in the elimination of positive charges of lysines and a relaxed chromatin conformation and mostly associated with increased transcriptional activity. On the other hand, histone deacetylases (HDACs) remove the acetyl group from the lysine residues in the N‐terminal tails of nucleosomal core histones, mainly resulting in a more compact chromatin conformation and repression of gene transcription. To date, in humans and mice, four classes of HDAC have been characterized, including class I (HDAC1, 2, 3, 8), class IIa (HDAC4, 5, 7, 9), class IIb (HDAC6, 10) and class IV (HDAC11).^15^ The class I members show homology to yeast RPD3 and normally localized in the nucleus. The class II HDACs share similarity with yeast HDA1 and have C‐terminal nuclear export signal, with the class IIa possessing myocyte enhancer factor (MEF)2–interacting domain at the N‐terminus, and the class IIb possessing tandem deacetylase domains. The class IV is unique in that it only has deacetylase domain. Accumulating evidence suggests the involvement of Hdacs in driving macrophage differentiation. For instance, in *Hdac3* knockout (KO) macrophages, almost half of the inflammatory genes, such as *Ifnb1‐* and Stat1‐dependent genes, fail to be activated in response to LPS, and inflammatory response is ameliorated.^16^, ^17^ Furthermore, *Hdac3* ^−/−^ macrophages show an IL4‐induced M2 phenotype and are hypersensitive to IL4 stimulation, due to the release of deacetylation at regulatory loci of many IL4‐regulated genes.^17^ Despite the epigenetic regulatory mechanisms in macrophage polarization, information regarding dMϕs in the local environment at the maternal‐foetal interface is limited.

In the present study, we have separated uterine CD45^+^F4/80^+^CD206^−^ M1‐like (M1) and CD45^+^F4/80^+^CD206^+^ M2‐like (M2) subpopulations by fluorescence‐activated cell sorting (FACS) during early pregnancy in mice. M1 preponderance was relevant to LPS‐induced abortion, which was rescued by adoptive transfer of M2. *Hdac9* was significantly up‐regulated in M2 in five sets of M1/M2 populations, including in vivo FACS‐sorted uterine and splenic macrophages, and in vitro peritoneal, bone marrow–derived and Raw 264.7 macrophages. Furthermore, *Hdac9* KO by CRISPR/Cas9 in Raw 264.7 and THP‐1 cells resulted in an exaggerated M2 response. Our findings reveal the function of Hdac9 in M1‐M2 balance and might provide insight into the understanding of macrophage‐based immunologic mechanisms relevant to abortion.

## 2 MATERIALS AND METHODS

### 2.1 Mice

Specific pathogen–free (SPF) inbred BALB/c mice at 8‐10 weeks were purchased from Beijing Vital River Laboratory Animal Technology Co., Ltd. (VRL, Beijing, China). The mice were bred and maintained in a temperature‐ and humidity‐controlled room with a constant photoperiod (light/dark = 12:12 hours). The protocols for animal studies were approved by the Committee on the Ethics of Animal Experiments of the Institute of Zoology, Chinese Academy of Sciences. Pregnancy was achieved by caging female mice with a male mouse at a 2:1 ratio, and the day when a copulatory plug was found was referred to as gestational day 1 (gd1).

### 2.2 Establishment of a low‐dose LPS‐induced mouse abortion model

Mice on gd6 were injected intraperitoneally (ip) with LPS (L3024, Sigma‐Aldrich) at 0.5, 1.2, 1.5, 2 or 4 μg reconstituted in 100 μL of phosphate‐buffered saline (PBS) per mouse (n = 3 in each group). At 24 hours after LPS administration, mice were killed by cervical dislocation and abortion was evaluated. The status of the foetuses was considered as death (abnormally shaped or haemorrhagic sacs) or resorption (small or pale sacs with no discernible foetus). The minimum dose of LPS that caused over 80% of abortion rate was determined. Subsequently, mice on gd6 were ip injected with the minimum dose of LPS and killed at 6, 12, 16, 18, 21, 24 and 30 hours thereafter. The time point at which over 80% of abortion rate was observed was determined as the most optimal time for uterine tissue sampling.

### 2.3 Isolation of mouse primary splenic and uterine macrophage cells

Primary splenic and uterine macrophage cells were freshly isolated from BALB/c mice. Briefly, spleens or uteri were removed from abdominal or uterine cavity, rinsed with PBS and then minced into small pieces. Minced uteri were placed in Hank's balanced salt solution (HBSS) buffer containing 0.3 mg/mL hyaluronidase (H3506, Sigma‐Aldrich), 1 mg/mL collagenase type IV (C5138, Sigma‐Aldrich) and 1 mg/mL BSA (36101ES60, Yeasen Biotechnology) for 25 minutes at 37°C. Red cell lysis buffer was used to remove red cells. The cell suspension was filtered through a 40‐μm nylon strainer, washed and re‐suspended with PBS containing 0.2% BSA. Single‐cell suspensions were incubated with anti‐mouse CD16/CD32 mAb (14‐0161‐85, eBioscience) for 10 minutes at 4°C to block Fc receptors and subsequently stained with PerCP‐Cyanine5.5–conjugated anti‐CD45 (Clone 30‐F11, eBioscience), PE‐conjugated anti‐F4/80 (Clone BM8, eBioscience) and APC‐conjugated anti‐CD206 (Clone 857615, R&D Systems) antibodies for 30 minutes at 4°C. After staining, cells were washed twice with FACS buffer (PBS with 2% FBS) and subjected to FACS sorting of macrophage cells. Data were acquired using a FACSCalibur flow cytometer (BD Biosciences) and analysed by FlowJo 7.6.1 software (Tree Star).

For flow cytometric analysis of macrophage subpopulations, an alternative APC‐conjugated anti‐mouse CD206 antibody (Clone C068C2, Biolegend) was used, and intracellular staining was performed after fixation with paraformaldehyde (PFA) (00‐8222‐49, eBioscience) and permeabilization with permeabilization buffer (00‐8333‐56, eBioscience).

### 2.4 In vivo adoptive transfer of splenic macrophage cells

Splenic macrophage cells were freshly sorted by FACS as described above. M1 and M2 macrophages were defined as CD45^+^F4/80^+^CD206^−^ and CD45^+^F4/80^+^CD206^+^ subpopulations, respectively. Each mouse on gd3 received 1 × 10^6^ M1 or M2 macrophage cells suspended in 100 μL of PBS by tail vein injection.

### 2.5 RNA sequencing and differential expression analysis

Uterine CD45^+^F4/80^+^CD206^−^ M1 and CD45^+^F4/80^+^CD206^+^ M2 macrophage cells from gd6 mice were FACS‐sorted. 1 × 10^6^ cells were collected, and total RNA was extracted using TRIzol reagent (15596018, Thermo Fisher Scientific). The qualities of the RNA samples were evaluated using the Agilent 2100 Bioanalyzer. RNA‐seq libraries were constructed from 1 μg of intact total RNA using NEBNext Ultra RNA Library Prep Kit for Illumina (#E7530L, NEB). The libraries were sequenced on an Illumina HiSeq 2500 platform as paired‐end 150 bp reads at Annoroad Gene Technology (Beijing, China; http://www.annoroad.com). Each step was strictly in accordance with transcriptome sequencing criteria.

After filtering low‐quality reads and those containing adapters with Trimmomatic, HISAT2 was used for building the genome index, and clean data were then mapped to the reference genome with default parameters. The reference genomes and the annotation file were downloaded from ENSEMBL database (http://www.ensembl.org/index.html). Read count for each gene in each sample was counted by HTSeq, and the abundance of each transcript in each sample was defined by FPKM (fragments per kilobase per million mapped reads). Sequencing results of two biologically repeated transcriptomes were synthetically analysed. DESeq2 was used for differential gene expression analysis. Genes with |log2FC| ≥ 1 and q < 0.05 were identified as differentially expressed genes (DEGs). Hierarchical clustering was applied to cluster DEGs. Genes were clustered together by different distance calculated by log2FPKM of each gene. The enrichment of genes in gene ontology (GO) terms compared with the background genes and of DEGs in Kyoto Encyclopedia of Genes and Genomes (KEGG) pathway between the two uterine macrophage subsets was implemented by Fisher's exact test, in which *P*‐value was adjusted by multiple comparisons as *q*‐value. GO terms or KEGG terms with *q* < 0.05 were considered to be significantly enriched.

### 2.6 Isolation of murine peritoneal macrophages (PM) and bone marrow–derived macrophages (BMM), RAW 264.7 cell culture and M1/M2 polarization

The BALB/c mice were killed by cervical dislocation and shortly immersed in 70% ethanol for sterilization. After injecting 15 mL PBS into the peritoneal cavity, PM cells were collected, washed with ice‐cold PBS and cultured in DMEM complete medium for 4 hours in a humidified 37°C and 5% CO_2_ incubator to obtain M0 cells.^18^

The bone marrow cells were flushed from femurs and tibia of BALB/c mice, collected and cultured with DMEM complete medium for 3 hours. The non‐adherent cells were collected and treated with 50 ng/mL of M‐CSF for 5 days. Medium was changed every 2 or 3 days to generate mature BMM at the M0 state.^19^

Mouse RAW 264.7 macrophage cells (TIB‐71, ATCC) were seeded onto a 6‐well plate (1.6 × 10^5^ cells/well) in Dulbecco's modified Eagle's medium (DMEM; SH30243, HyClone) supplemented with 10% heat‐inactivated foetal bovine serum (Thermo Fisher Scientific), 100 U/mL penicillin and 100 µg/mL streptomycin.

To differentiate cells into M1 macrophages, the cytokines used were LPS (100 ng/mL; L3024, Sigma‐Aldrich) and IFNG (20 ng/mL; 315‐05, PeproTech). For M2 macrophage differentiation, cells were stimulated with IL4 (214‐14, PeproTech) and IL13 (210‐13, PeproTech) at a concentration of 20 ng/mL. The treatment time was optimized as 24 hours.

### 2.7 THP‐1 cell culture, macrophage differentiation and polarization

Human monocyte THP‐1 cells (TIB‐202, ATCC) were maintained in culture in RPMI 1640 medium (SH30027, HyClone) containing 10% of heat‐inactivated foetal bovine serum, 100 U/mL penicillin and 100 µg/mL streptomycin and 50 pmol/L β‐mercaptoethanol. THP‐1 monocytes are differentiated into macrophage‐like cells (THP‐1 macrophages) by 24‐hour incubation with 160 nmol/L phorbol 12‐myristate 13‐acetate (PMA; P8139, Sigma‐Aldrich) followed by 24‐hour resting in PMA‐free medium. The 24‐hour incubation time with PMA was determined by comparing the expression of macrophage surface markers CD68 and CD14 between 24 and 48‐hour treatments, and there was no apparent difference between the two time points (Figure 3A, right panel). THP‐1 macrophages were polarized to M1 states by incubation with 100 ng/mL of LPS (L3024, Sigma‐Aldrich) and 20 ng/mL of IFNG (300‐02, PeproTech) for 24 hours. M2 macrophages were obtained by incubation with 20 ng/mL each of IL4 (200‐04, PeproTech )and IL13 (200‐13, PeproTech) for 24 hours.

### 2.8 RNA isolation and real‐time quantitative reverse transcription PCR (qRT‐PCR) analysis

Total RNA was extracted using TRIzol reagent, and reverse transcription was performed with 1 µg RNA and M‐MLV reverse transcriptase (28025‐021, Thermo Fisher Scientific) according to the manufacturer's instructions. cDNA was subjected to quantitative real‐time PCR using primers listed in Table S1 and UltraSYBR Mixture (CW0957 M, CWBIO, Beijing, China) on a Light Cycler 480 real‐time PCR System (Roche). Cycle threshold values were normalized to the housekeeping gene *Gapdh,* and 2^−ΔΔCT^ was used to calculate changes in relative mRNA expression between groups. Normalized expression levels are displayed as means ± SEM (standard error of the mean) of at least three replicates.

### 2.9 Generation of *Hdac9*/*HDAC9* KO RAW 264.7 and THP‐1 cell lines

CRISPR/Cas9 technology was utilized to generate *Hdac9*/*HDAC9* KO RAW 264.7 and THP‐1 cell lines, respectively. Guide RNAs (gRNAs) flanking the target exon close to the N‐terminus and resulting in frameshift mutations were designed using the CRISPR design tool (http://CRISPR.mit.edu). For mouse *Hdac9* gene, the common N‐terminal 242‐bp exon (RefSeq NM_024124.3) of all transcript variants was targeted. For human *HDAC9* gene, the common N‐terminal 142/151‐bp exon of 39 transcript isoforms (the third exon of transcript variant 1; NM_058176) was targeted. All the target exons were first confirmed to be expressed before performing the KO experiments. Guide RNA sequences (Table S2) were cloned into the pSpCas9 (BB)‐2A‐GFP (PX458) plasmid (#48138, Addgene) and validated by sequencing. Empty PX458 vector or pairs of gRNA‐PX458 constructs were transfected into RAW 264.7 cells with Lipofectamine LTX (A12621, Thermo Fisher Scientific) or electro‐transfected into THP‐1 cells in the Gene Pulser Xcell system (Bio‐Rad) at 220 Volt with 950 μ Farad capacitance and infinite resistance in a 4‐mm cuvette, respectively, according to the manufacturer's instructions. Two days after transfection, GFP^+^ single cells were sorted into 96‐well plates by flow cytometry. Homozygous KO clones were confirmed by genomic PCR genotyping using primers spanning the deleted exon followed by DNA sequencing of PCR products directly or after T‐vector cloning, by qRT‐PCR using primers (Table S2) within or spanning the deleted exon, and/or by immunoblotting analysis. Three or four independent vector control or KO single‐cell clones were analysed for each experiment.

### 2.10 Western blot analysis

Cells were lysed in RIPA buffer (C1053, APPLYGEN, Shanghai, China) with 1 mmol/L phenylmethanesulphonyl fluoride (PMSF) for 30 minutes on a rocker at 4°C, and protein concentration was quantified by using the Pierce BCA Protein Assay Kit (23227, Thermo Fisher Scientific). Proteins were separated by SDS‐PAGE and transferred onto a nitrocellulose membrane (Pall). The membranes were blocked in 5% skimmed milk in TBST at 37°C for 1 hour and then incubated with anti‐HDAC9 (PA5‐42247, Thermo Fisher Scientific) at 4°C overnight, followed by incubation with secondary antibodies conjugated to HRP (074‐1506, KPL) for 1 hour at room temperature. The results were visualized with a Gene Gnome XRQ Chemiluminescence detector (Syngene) and GAPDH was used as a protein loading control.

### 2.11 MTT assay

Cells were seeded in 96‐well plates at an optimal concentration and cultured for the indicated time periods. 15 μL 5 mg/mL of MTT reagent (M8180, Solarbio, Beijing, China) was added into each well, followed by an additional 4 hours of incubation at 37 C. The formazan blue product that formed in the cells was dissolved by adding 150 μL of dimethyl sulphoxide (DMSO). The optical density was measured at 570 nm. Data are expressed as means ± SEM of four replicate measurements.

### 2.12 Measurement of NO production in RAW 264.7 M1 macrophage cells

RAW 264.7 cells were seeded into 96‐well plates and polarized into M1 macrophages for 24 hours as indicated above. The NO concentrations were measured using Griess reagent (S0021, Beyotime, Shanghai, China) by evaluating the released nitrite content in the cell supernatant. The OD values were determined at 540 nm using a microplate reader, and data are expressed as means ± SEM of five replicates.

### 2.13 Phagocytosis assay

Cells were incubated with carboxylate‐modified fluorescent latex beads with a mean diameter of 2 μm (L3030; Sigma‐Aldrich, 1:400 dilution) for 4 hours, as described previously.^20^ After incubation, the supernatant was discarded and the cells were trypsinized and washed three times with ice‐cold PBS. Cells were fixed with 4% formaldehyde, and the percentage of intracellular fluorescent beads was analysed on a BD FACSCalibur flow cytometer.

For live‐cell imaging, cells were plated in Nunc Lab‐Tek 8‐well chamber slides (Thermo Fisher Scientific) and time‐lapse image acquisitions were performed through the PerkinElmer precisely UltraVIEWVoX3D live‐cell imaging system (PerkinElmer) equipped with a 37°C incubator and 5% CO_2_ supply. Images were captured every 2 seconds for 60 minutes with a z‐resolution of 2.0 μm at 20× magnification and were analysed using Volocity software (PerkinElmer).

### 2.14 Statistical analysis

All experiments were repeated as indicated in the figure legends, and n indicates the number of independent biological repeats. Data are presented as the means ± SEM. Student's t test was used to evaluate the differences between two groups, and analysis of variance (ANOVA) was performed when more than two groups were compared. *P < .05; **P < .01. For all statistical tests, *P* < .05 was considered statistically significant.

## 3 RESULTS

### 3.1 The dynamic balance of M1 and M2 uterine macrophages during mouse early pregnancy was disrupted after LPS treatment

**FIGURE 1:**
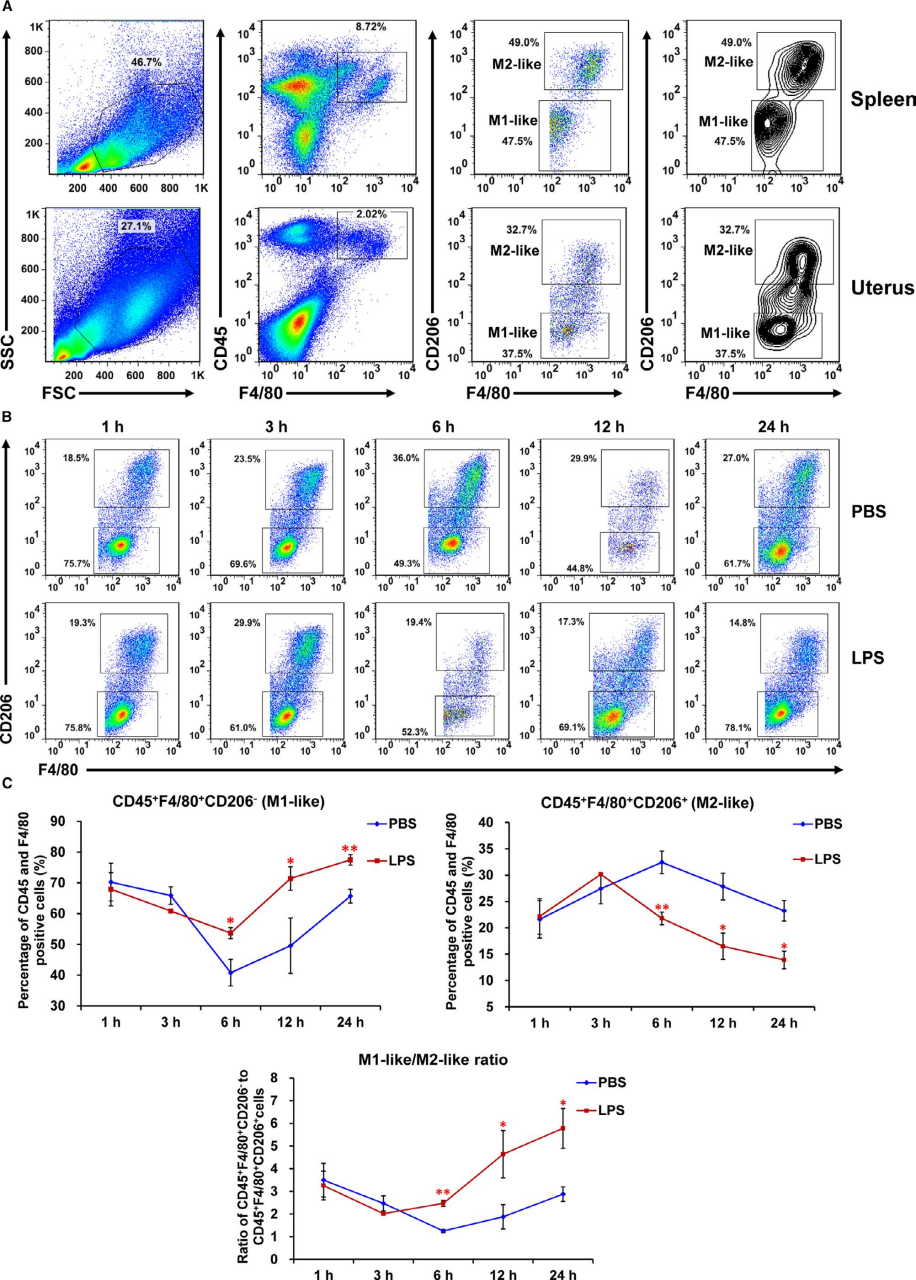
Flow cytometry analysis of uterus M1 and M2 macrophages and dynamic changes of two macrophage subsets after treatment with LPS. A, Gating strategy used to identify M1‐like (CD45^+^F4/80^+^CD206^−^ cells) and M2‐like (CD45^+^F4/80^+^CD206^+^ cells) macrophages in uterine and splenic tissues from BALB/c mouse on gd6. Total leucocytes (CD45^+^ cells) were gated within the leucocyte gate using forward light scatter (FSC) versus side scatter (SSC). B, The proportions of the two macrophage subsets at 1, 3, 6, 12 and 24 h after LPS (0.5 µg/100 µL PBS) ip injection on gd6 by flow cytometry analysis. C, Statistical analysis of the percentages of M1 or M2 cells, and ratio of M1/M2 macrophages at 1, 3, 6, 12 and 24 h following LPS or PBS treatment (experimental data shown in panel B). Data are shown as line graphs (means ± SEM. **P < .01, *P < .05, n = 3)

As mentioned, mouse dMϕs are predominantly M1 phenotype during embryo implantation and switched to a balanced M1/M2 profile afterwards. We initiated to choose gd6, the end of the putative peri‐implantation window, to distinguish mouse uterine macrophage subpopulations using flow cytometry. According to the literature, we utilized different staining strategies with antibodies against various surface markers, such as CD45, F4/80, CD206, CD11c and MHCII. As a result, using PerCp‐Cy5.5‐conjugated anti‐mouse CD45, PE‐conjugated anti‐mouse F4/80 and APC‐conjugated anti‐mouse CD206 antibodies, CD45^+^F4/80^+^CD206^−^ M1 and CD45^+^F4/80^+^CD206^+^ M2 macrophage subpopulations were identified both in the spleen and the uterus on gd6 (Figure 1A). Next, we used a low‐dose LPS‐induced abortion model (0.5 μg per mouse)^21^, ^22^ to investigate the dynamic changes of M1 and M2 macrophages after LPS treatment for 1, 3, 6, 12 and 24 hours. The results showed an overall increasing tendency of M1 cells, a decreasing trend of M2 cells and an increase in M1/M2 ratio, and significant differences between LPS‐injected mice and the control group were observed at 6 hours and afterwards (Figure 1B,C, and Figure S1). This indicates that M1 macrophages are the predominant phenotype in the decidua following treatment with LPS, and this imbalance occurs before abortion.

### 3.2 The adoptive transfer of M2 macrophages can effectively rescue LPS‐induced abortion

**FIGURE 2:**
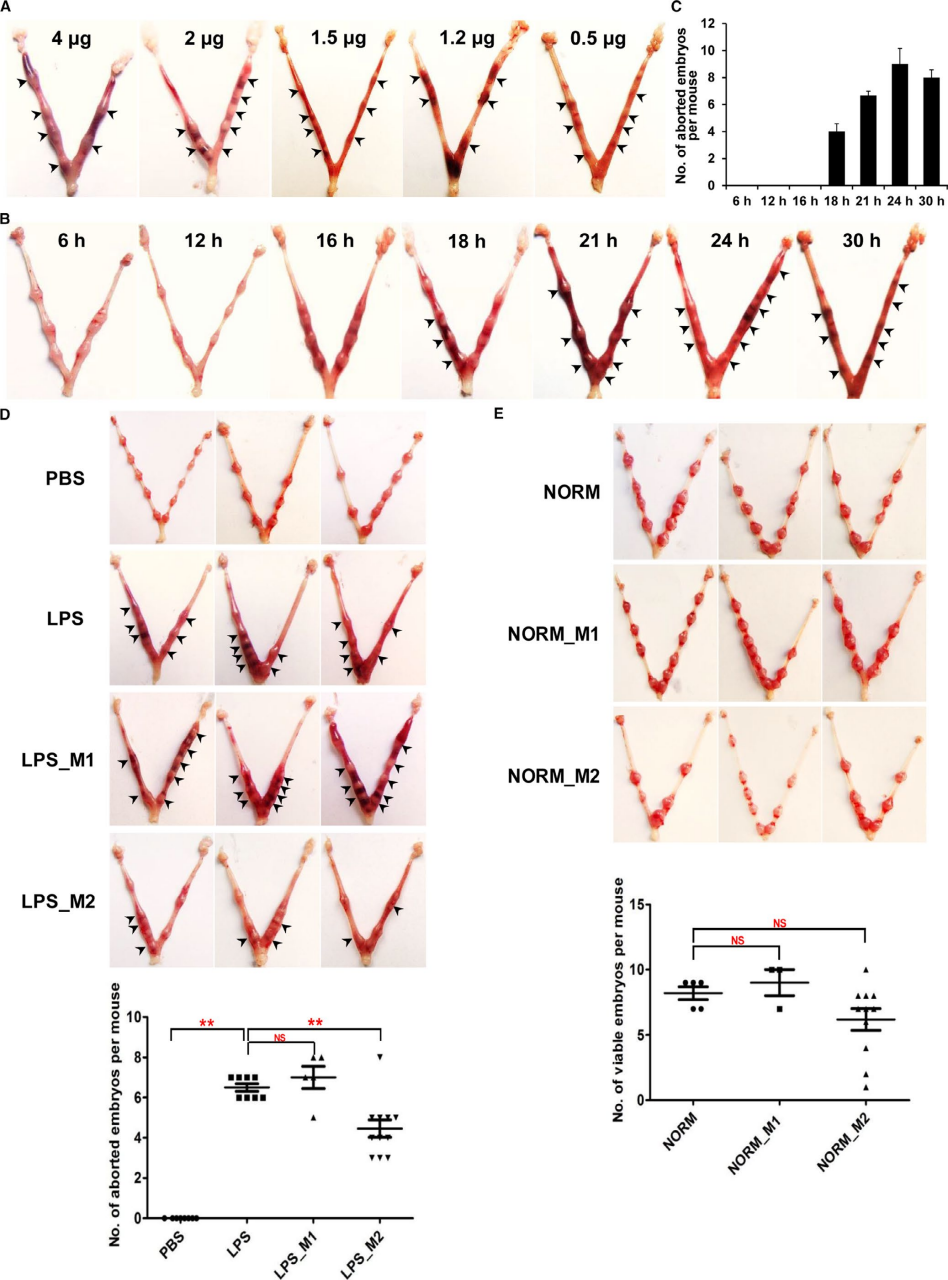
Effects of adoptive transfer of M1 or M2 cells on low‐dose LPS‐induced abortion or normal pregnancy in BALB/c mice. A, Different doses of LPS (0.5, 1.2, 1.5, 2 or 4 μg) were ip injected on gd6. The abortion was observed at 24 h post‐LPS treatment. B, Each mouse was given 0.5 μg LPS on gd6, and the abortion was analysed at 6, 12, 16, 18, 21, 24 and 30 h after LPS administration. C, Statistical analysis of the number of aborted embryos at different time points. D, Effect of adoptive transfer of M1 or M2 macrophage on early abortion induced by LPS. Representative phenotypes are shown. 1 × 10^6^ M1 and M2 macrophages cells were transferred on gd3 via tail vein injection, and 0.5 µg LPS was administrated on gd6. A same volume of PBS was given to control groups. Uteri were collected 21 h after LPS or PBS injection. E, Representative phenotypes of adoptive transfer of M1 or M2 cells into normal pregnant mice. NORM, normal pregnancy; NORM_M1, normal pregnancy with adoptive M1 transfer; NORM_M2, normal pregnancy with adoptive M2 transfer. In (D and E), the statistical analysis of the number of aborted embryos was shown below (data were expressed as means ± SEM. **P < .01). Arrows indicate the site of abortion

In order to study the abortion mechanism caused by M1/M2 imbalance, rescue experiments utilizing in vivo LPS‐induced abortion model are needed. Since high as well as low doses of LPS can induce abortion in mice,^22^ we set up to optimize the treatment conditions. Mice were ip injected with different doses of LPS (0.5, 1.2, 1.5, 2 or 4 μg in 100 μL PBS per mouse) on gd6. After 24 hours, we observed that 0.5 μg of LPS was the lowest effective dose in inducing abortion (Figure 2A). Meanwhile, at 0.5 μg dose of LPS, different treatment time was set (6, 12, 16, 18, 21, 24 and 30 hours). According to the analysis of the number of aborted embryos, the mice showed obvious signs of abortion at 21 hours (Figure 2B,C). Therefore, treatment with 0.5 μg of LPS for 21 hours was utilized for later adoptive transfer experiments.

Next, we isolated splenic M1 and M2 macrophage cells from 6‐ to 8‐week‐old female BALB/c mice and transferred 1 million of M1 or M2 cells to Day 3 pregnant BALB/c mice. On gd6, 0.5 μg/100 μL LPS was injected, and the same dose of PBS was injected into the control group. Notably, LPS‐induced abortion after M2 transfer was significantly recovered compared with the control group that received LPS alone, but M1 macrophages failed to exaggerate abortion (Figure 2D). In parallel, tail intravenous injection of the same amount of M1 or M2 macrophages had no effects in normal pregnant mice, presumably because the dose of macrophages was insufficient to reverse or to change pregnancy outcome (Figure 2E).

### 3.3 Establishment of mouse and human M1‐ and M2‐type macrophage polarization models in vitro

**FIGURE 3:**
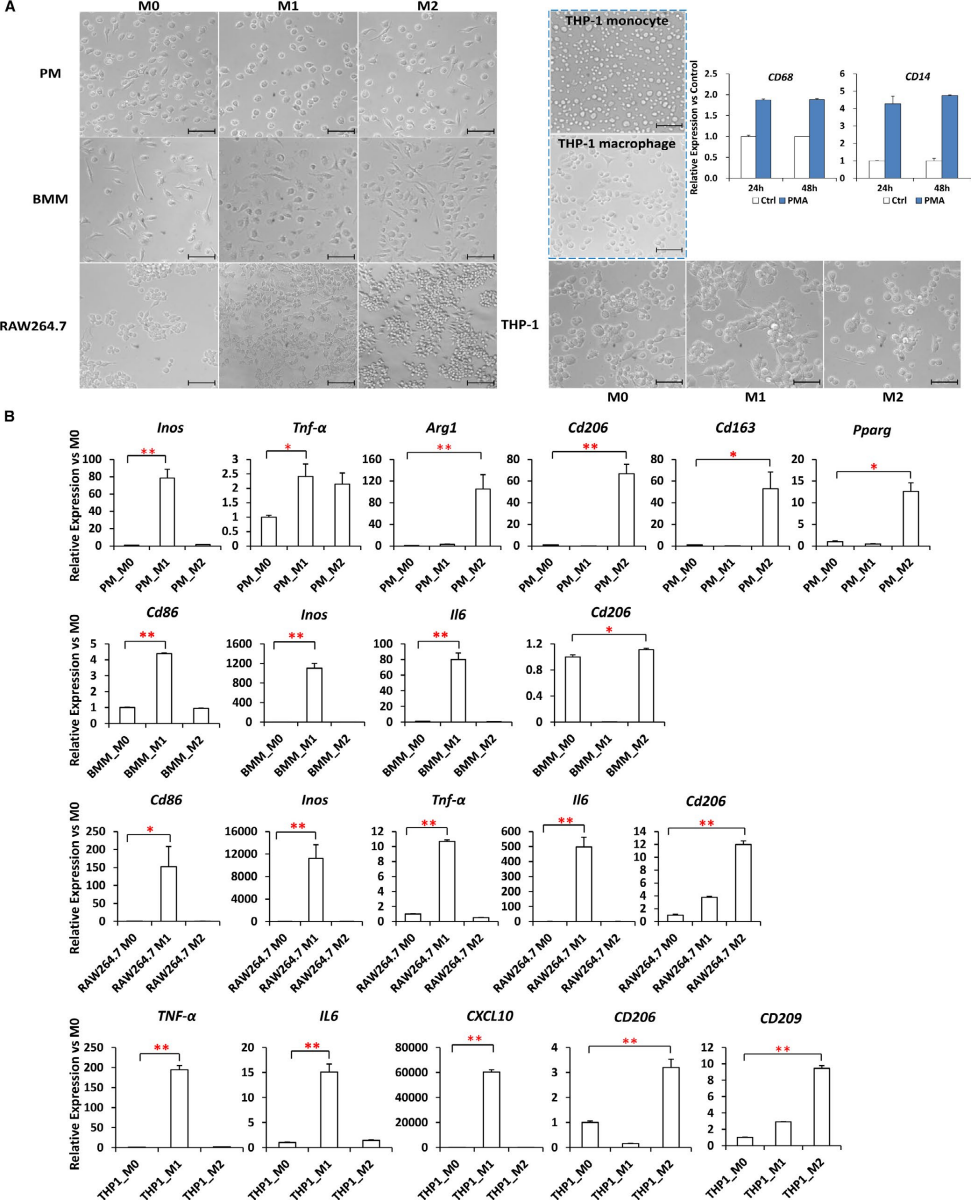
Establishment of in vitro M1/M2 polarization models in mouse PM, BMM, RAW 264.7 cell line and human THP‐1 monocyte–derived macrophages. A, Cells were classically activated to M1 condition with 100 ng/mL LPS + 20 ng/mL IFNG for 24 h and alternatively activated to M2 condition with 20 ng/mL IL4 + IL13 for 24 h, respectively. Morphological changes were recorded by light microscopy. For THP‐1 monocytes, cells were firstly differentiated into macrophages by 24‐ or 48‐h treatment with 160 nmol/L PMA and a recovery for another 24 h with fresh medium without PMA. The expression of macrophage surface markers *CD68* and *CD14* was determined by qRT‐PCR. Scale bars, 100 μm. B, The expression of M1 markers (*Inos* and *Tnf‐α* in PM; *Cd86*, *Inos* and *Il6* in BM; *Cd86*, *Inos*, *Tnf‐α* and *Il6* in RAW 264.7 cells; *TNF‐α*, *IL6* and *CXCL10* in THP‐1 cells) and M2 markers (*Arg1*, *Cd206*, *Cd163* and *Pparg* in PM; *Cd206* in BM; *Cd206* in RAW 264.7 cells; *CD206* and *CD209* in THP‐1 cells) after induced polarization by qRT‐PCR (means ± SEM. **P < .01, *P < .05, n = 3)

In order to further study macrophage M1/M2 polarization in vitro, we utilized mouse PM, BMM, RAW 264.7 macrophage cell line and human THP‐1 monocyte–derived macrophage cell line to establish mouse and human M1/M2 macrophage polarization models in vitro (Figure 3A). THP‐1 monocytes were firstly induced to differentiate into macrophages by PMA treatment, as evidenced by increased expression of macrophage surface markers *CD68* and *CD14* (Figure 3A, right panel).^23^ qRT‐PCR analysis showed that M1 and M2 differentiation markers were significantly up‐regulated in these four models. In detail, the M1 markers include the following: *Inos* and *Tnf‐α* in PM; *Cd86*, *Inos* and *Il6* in BMM; *Cd86*, *Inos*, *Tnf‐α* and *Il6* in RAW 264.7 cells; *TNF‐α*, *IL6* and *CXCL10* in THP‐1 cells. The M2 markers include the following: *Arg1*, *Cd206*, *Cd163* and *Pparg* in PM; *Cd206* in BMM; *Cd206* in RAW 264.7 cells; *CD206* and *CD209* in THP‐1 cells (Figure 3B).

**FIGURE 4:**
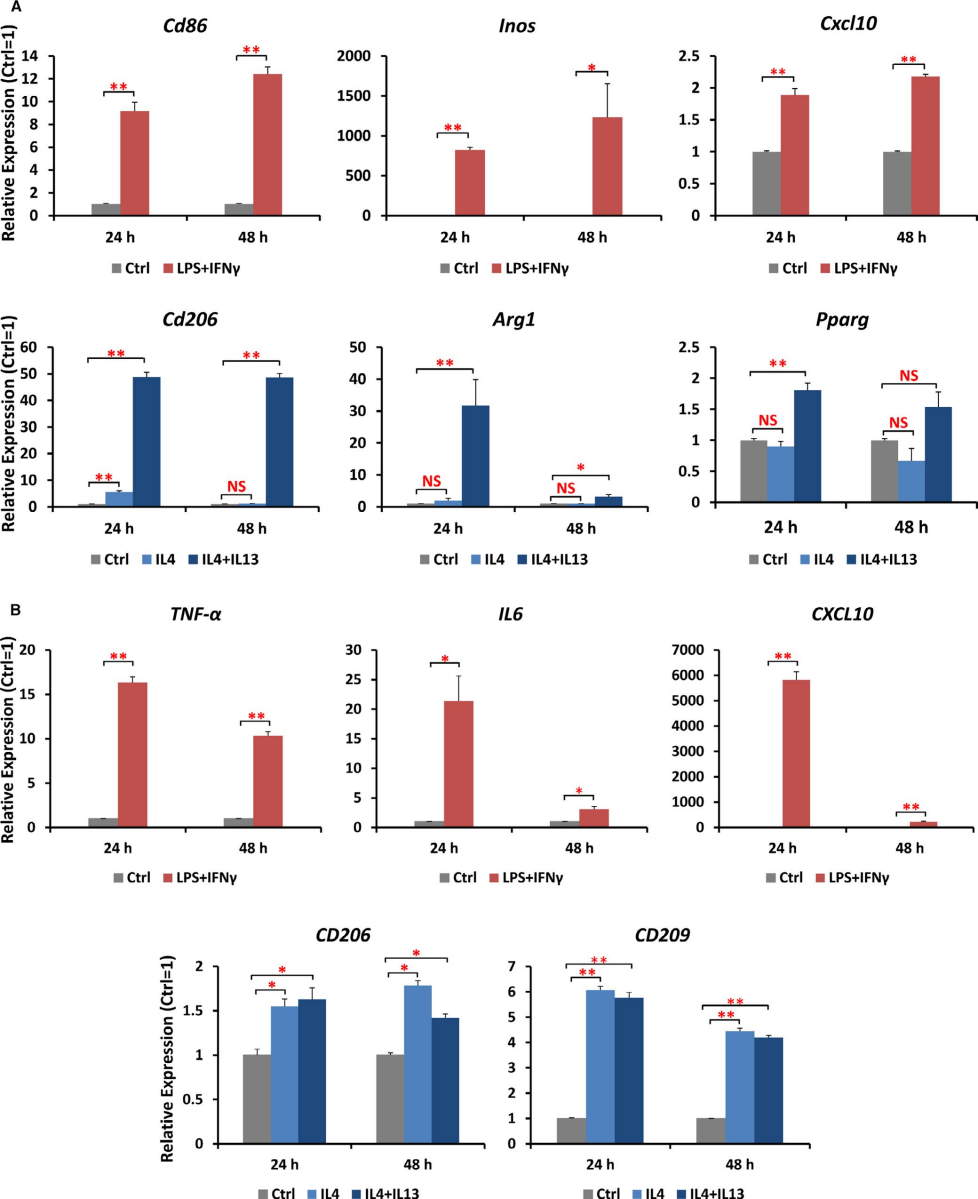
Expression of M1/M2 polarization markers under different treatment conditions for different time were validated by qRT‐PCR. Cells were activated to M1 condition with 100 ng/mL LPS + 20 ng/mL IFNG, or activated to M2 condition with 20 ng/mL IL4 or 20 ng/mL dual IL4 + IL13 for 24 and 48 h, respectively. Cells that received media alone were defined as M0 condition. A, The mRNA expression of M1 markers *Cd86*, *Inos*, *Cxcl10* and M2 markers *Cd206*, *Arg1*, *Pparg* in RAW 264.7 cells. B, The mRNA expression of M1 markers *TNF‐α*, *IL6*, *CXCL10* and M2 markers *CD206* and *CD209* in THP‐1‐derived macrophages. Data were expressed as means ± SEM. **P < .01, *P < .05, n = 3

The in vitro M1/M2 polarization models in the literature showed inconsistent cytokine treatment doses and duration. To obtain optimized polarization conditions, RAW 264.7 cells and THP‐1‐derived macrophage cells were stimulated with LPS+IFNG to M1 or IL4/IL4+IL13 to M2 for 24 and 48 hours, respectively. The mRNA expression of M1 markers was profoundly up‐regulated after 24 hours of treatment, and either remained relatively stable (in RAW 264.7 cells) or down‐regulated (in THP‐1 cells) at 48 hours (Figure 4A,B). Expression of M2 markers was significantly increased at 24‐hour treatment in response to double IL4/IL13 cytokines and was kept at a similar level or even decreased at 48 hours in Raw 264.7 cells (Figure 4A). However, up‐regulation of these marker genes was not satisfactory upon treatment with IL4 only, inconsistent with the results from previous studies.^24^, ^25^ In THP‐1 cells, expression profiles of M2 markers did not show any obvious difference either between single and double cytokines, or between different induction time (Figure 4B). Therefore, in the following studies, M1‐ and M2‐type macrophage polarization in RAW 264.7 and THP‐1 cells was established by stimulation with 100 ng/mL LPS + 20 ng/mL IFNG, and with 20 ng/mL IL4 + 20 ng/mL IL13, respectively, for 24 hours.

### 3.4 RNA‐seq analysis of DEGs in mouse uterine M1 versus M2 on Day 6 of pregnancy (gd6)

We next adopted bulk RNA‐Seq to investigate DEGs between M1 and M2 macrophage subpopulations in mouse uterus on gd6. Figure S2A shows the FACS‐sorted M1 (CD45^+^F4/80^+^CD206^−^) and M2 macrophages (CD45^+^F4/80^+^CD206^+^) from gd6 mouse uteri. Two biological replicates were performed in both M1 (CD206‐negative, CD206N) and M2 groups (CD206‐positive, CD206P). The total reads and mapping efficiency of each sample are shown in Table S3, and the count of detected genes in each sample is illustrated in Figure S2B. We identified 1837 DEGs, among which 629 was up‐regulated in M2 versus M1 and 1208 was down‐regulated (Figure S2B, left panel). Heatmap hierarchical clustering analysis of DEGs confirmed similar gene expression patterns between the two replicates of each sample (Figure S2B, right panel).

GO enrichment analysis was used to investigate the biological functions of DEGs between M1 and M2. The majority of the responsive GO terms were found in the biological processes, followed by molecular functions and cellular components. The biological processes that were significantly enriched were mainly involved in regulation of multicellular organismal process, cell adhesion, cell surface receptor signalling pathway, biological adhesion, regulation of response to stimulus, positive regulation of biological process, regulation of multicellular organismal development, regulation of localization, regulation of developmental process and regulation of cellular component movement (Figure S2C). The top 10 enriched GO terms in molecular functions and cellular components are illustrated in Table S4 and S5.

Based on analyses of the up‐ and down‐regulated genes enriched in each gene categories, a total of 77 significantly enriched KEGG pathways (Table S6) were observed, including cytokine‐cytokine receptor interaction, cell adhesion molecules, haematopoietic cell lineage, complement and coagulation cascades, extracellular matrix‐receptor interaction, lysosome, and Th1 and Th2 cell differentiation. All of these are immune system‐related and among the top 10 mostly significant KEGG pathways.

Based on the literature and our previous data, we are interested with epigenetic regulation, in particular, histone modification mechanisms, during M1/M2 differentiation. By analysing the DEGs in all comparison groups, we found three members of Hdac family, *Hdac7*, *8* and *9*, were putative DEGs between M1 and M2 cells. As Hdacs are well‐known players in macrophage‐mediated host defence with abundant expression levels yet elusive mechanisms, this drives us to investigate the function of HDACs in uterine macrophage polarization.

### 3.5 Validation of the differential expression of *Hdac9* in uterine, splenic and in vitro polarized PM, BMM and Raw 264.7 M1/M2 cells using qRT‐PCR

**FIGURE 5:**
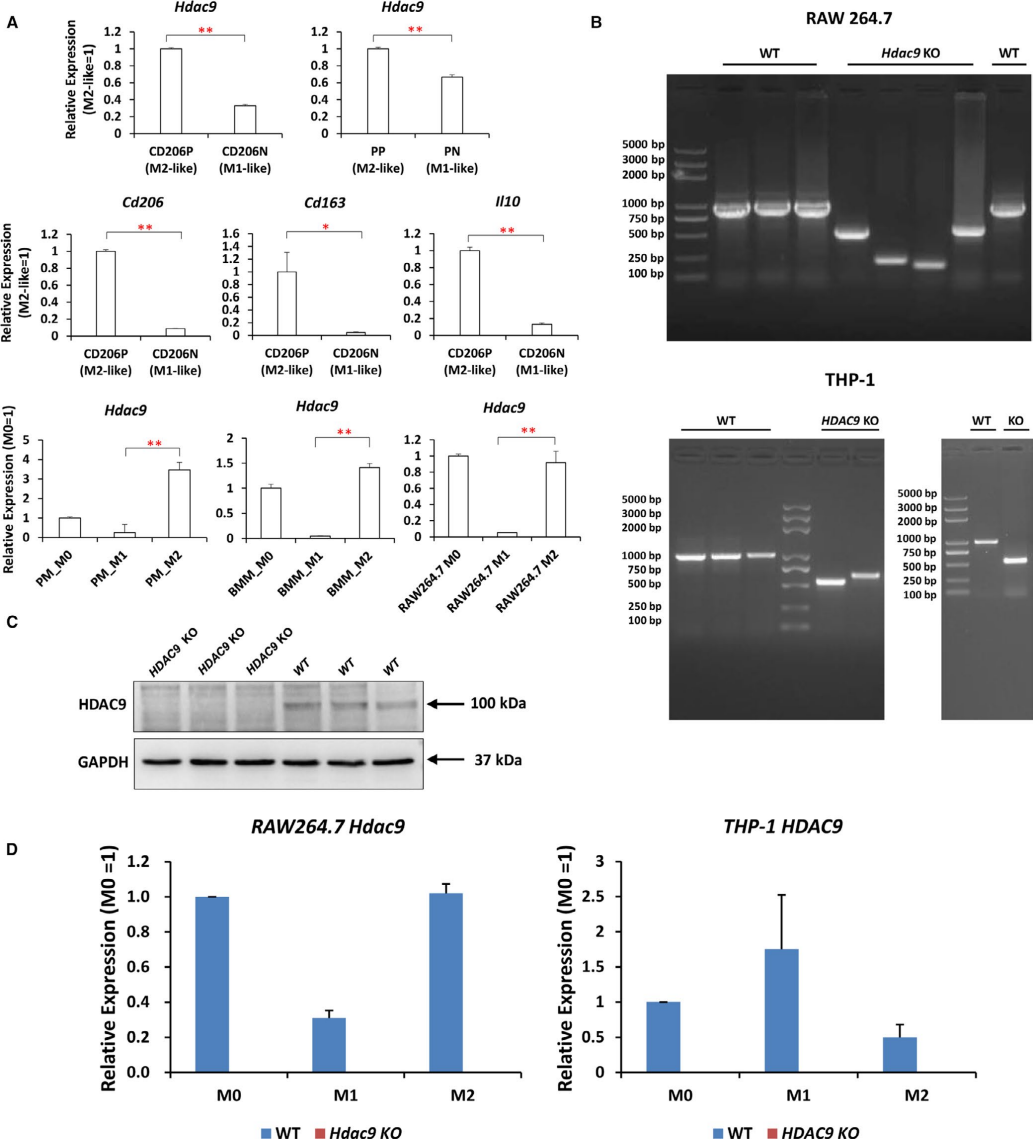
Generation of CRISPR/Cas9‐mediated *Hdac9*/*HDAC9* KO cell line in Raw 264.7 cells and THP‐1 cells. A, Higher expression of *Hdac9* in M2 cells as compared to their corresponding M1 cells, in FACS‐sorted uterine macrophages (CD206P vs. CD206N), splenic macrophages (PP vs. PN) and in vitro polarization models of mouse PM, BMM and RAW 264.7 cells. Data are expressed as means ± SEM (n = 3). **P < .01, *P < .05. Higher expression of M2 marker genes *Cd206*, *Cd163* and *Il10* was also confirmed in CD206P vs CD206N. B, Verification of *Hdac9*/*HDAC9* KO in Raw 264.7 and THP‐1 cells by using PCR primers spanning the target exons and PCR amplification from genomic DNA. C, Western blot analysis shows the absence of HDAC9 protein in THP‐1 cells. D, Confirmation of *Hdac9*/*HDAC9* KO in RAW 264.7 cells and THP‐1 cells at M0, M1 and M2 states by qRT‐PCR (means ± SEM. n = 3)

Next, we assessed the transcript levels of Hdacs and marker genes in FACS‐sorted gd6 uterine M1 and M2 cells, to validate the RNA‐Seq data. *Hdac9* was revealed to be significantly up‐regulated in gd6 M2 as compared to M1 by qRT‐PCR (Figure 5A, upper left panel). Meanwhile, the expression patterns of M2 marker genes *Cd206*, *Cd163* and *Il10* were also confirmed, similar to the sequencing data (Figure 5A, middle panel). M1 marker genes *Inos* and *Pparg* were not expressed, which may imply that uterine macrophages have different phenotypes from macrophages that originated from other tissues. We further utilized FACS‐sorted splenic M1 and M2 macrophages, M1/M2 polarized murine PM, BMM and Raw 264.7 cells and performed qRT‐PCR analysis. Profoundly, *Hdac9* showed consistent expression profiles in all the five sets of M1/M2 pairs (Figure 5A). However, *Hdac7* and *Hdac8* showed inconsistent or contradictory expression patterns in these five M1/M2 pairs (data not shown). We assume that as compared to other Hdac members, Hdac9 might play more fundamental and conserved roles in M1/M2 differentiation. Therefore, we anchored Hdac9 as our target interest gene.

### 3.6 *Hdac9* deficiency caused exaggerated M2 polarization in Raw 264.7 and THP‐1 cell line

To determine the conserved function of HDAC9 in mouse and human macrophage polarization, we performed CRISPR‐Cas9‐mediated *Hdac9*/*HDAC9* ablation in Raw 264.7 and THP‐1 cells. Genomic genotyping PCR (Figure 5B), qRT‐PCR (Figure 5D) and Western blot (Figure 5C) analysis confirmed successful homozygous KO in both cells. All the KO clones showed undetectable levels of transcript and/or protein expression (Figure 5C,D). We were unable to detect convincing immune‐reactive proteins using commercially available anti‐mouse HDAC9 antibodies. Overall, 4 single‐cell expanded homozygous KO clones were obtained at an efficiency of approximately 6.6% (4/61) in Raw 264.7 cells, and 3 single‐cell KO clones were obtained among 40 clones in THP‐1 cells (approximate efficiency of 7.5%).

### 3.7 *Hdac9*/*HDAC9* KO exaggerates expression of M2 marker genes in both Raw 264.7 cells and THP‐1‐derived macrophage cells

**FIGURE 6:**
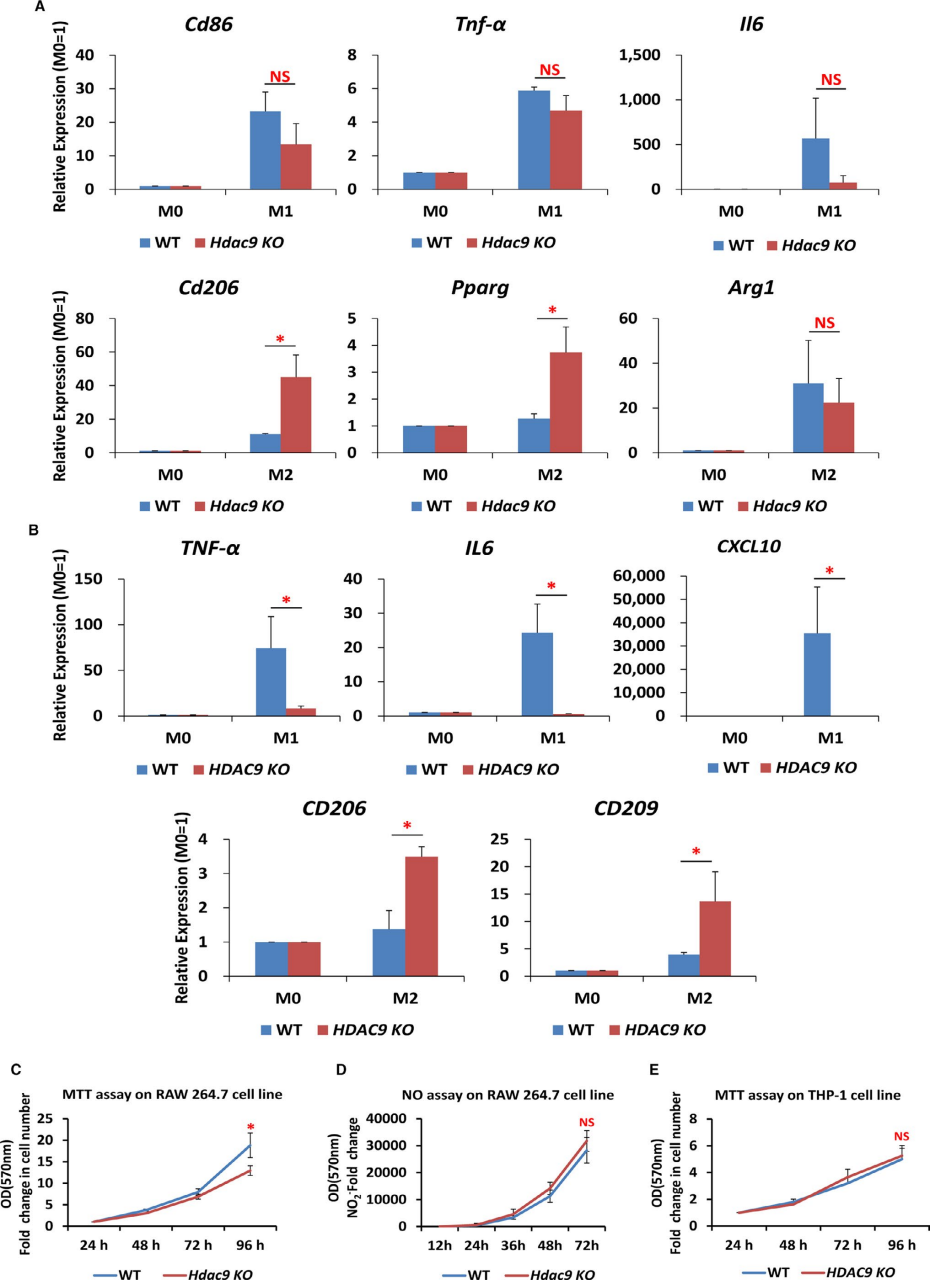
Effect of *Hdac9*/*HDAC9* ablation on the expression of M1/M2 markers, proliferation and NO production of macrophages. A, In Raw 264.7 cells, compared with WT controls, expression of M1 markers *Cd86*, *Tnf‐α* and *Il6* mRNA remained unchanged and M2 markers *Cd206* and *Pparg* were significantly increased in *Hdac9* KO cells. B, In THP‐1‐derived macrophages, compared with WT controls, expression of M1 markers *TNF‐α*, *IL6*, *CXCL10* were significantly down‐regulated, and M2 markers *CD206* and *CD209* were significantly up‐regulated in KO cells. n = 3 biological replicates per group. *P < .05. C, MTT assay of WT and *Hdac9* KO Raw 264.7 cells at 24, 48, 72 and 96 h of proliferation. D, Effect of *Hdac9* KO on LPS/IFNG‐induced NO production analysed by the Griess reaction assay in RAW 264.7 cells. E, MTT assay of WT and *HDAC9* KO THP‐1 cells at 24, 48, 72 and 96 h of proliferation. Values are means of five replicates (n = 5) ± SEM. *P < .05

We next performed qRT‐PCR to determine the expression level of M1‐ and M2‐type macrophage polarization markers within *Hdac9*/*HDAC9* KO Raw 264.7 or THP‐1‐derived macrophages. In Raw 264.7 cells, the *Hdac9* KO macrophages expressed significantly higher levels of M2 markers *Cd206* and *Pparg*, while the levels of M1 markers *Cd86, Tnf‐α* and *Il6* remained unchanged (Figure 6A). Interestingly, *HDAC9*‐deficient THP‐1‐derived macrophages expressed significantly higher levels of M2 markers *CD206* and *CD209*, whereas expressing lower levels of M1 markers *TNF‐α*, *IL6* and *CXCL10* (Figure 6B). Therefore, these observations implicated that *Hdac9*/*HDAC9* deficiency caused an up‐regulation of M2 markers and functioned as a repressor of the M2‐type macrophage polarization.

To exclude the possibility that the effect of *Hdac9* deficiency on M1/M2 marker gene expression was not an indirect reaction of proliferation abnormalities, we performed MTT assay to assess the cell viability at 24, 48, 72 and 96 hours. The results showed that the viability of *Hdac9* KO Raw 264.7 cells was significantly decreased only after 96 hours (Figure 6C) and that no effect was observed in THP‐1 cells (Figure 6E). Therefore, the exaggeration of the expression of M2 markers after *Hdac9* ablation was unlikely caused by a decreased proliferation or viability potential at the M0 state.

### 3.8 *Hdac9* KO had no effect on LPS/IFNG‐induced M1‐type pro‐inflammatory NO release, but increased the phagocytosis capacity of M2‐type macrophage in RAW 264.7 cells

We further performed NO assay and phagocytosis assay to detect functional phenotypes in *Hdac9* KO clones distinguishable from wild‐type (WT; empty vector) controls. There was no significant change in NO release after *Hdac9* KO compared with WT controls (Figure 6D). NO production in THP‐1‐differentiated macrophages was undetectable.

**FIGURE 7:**
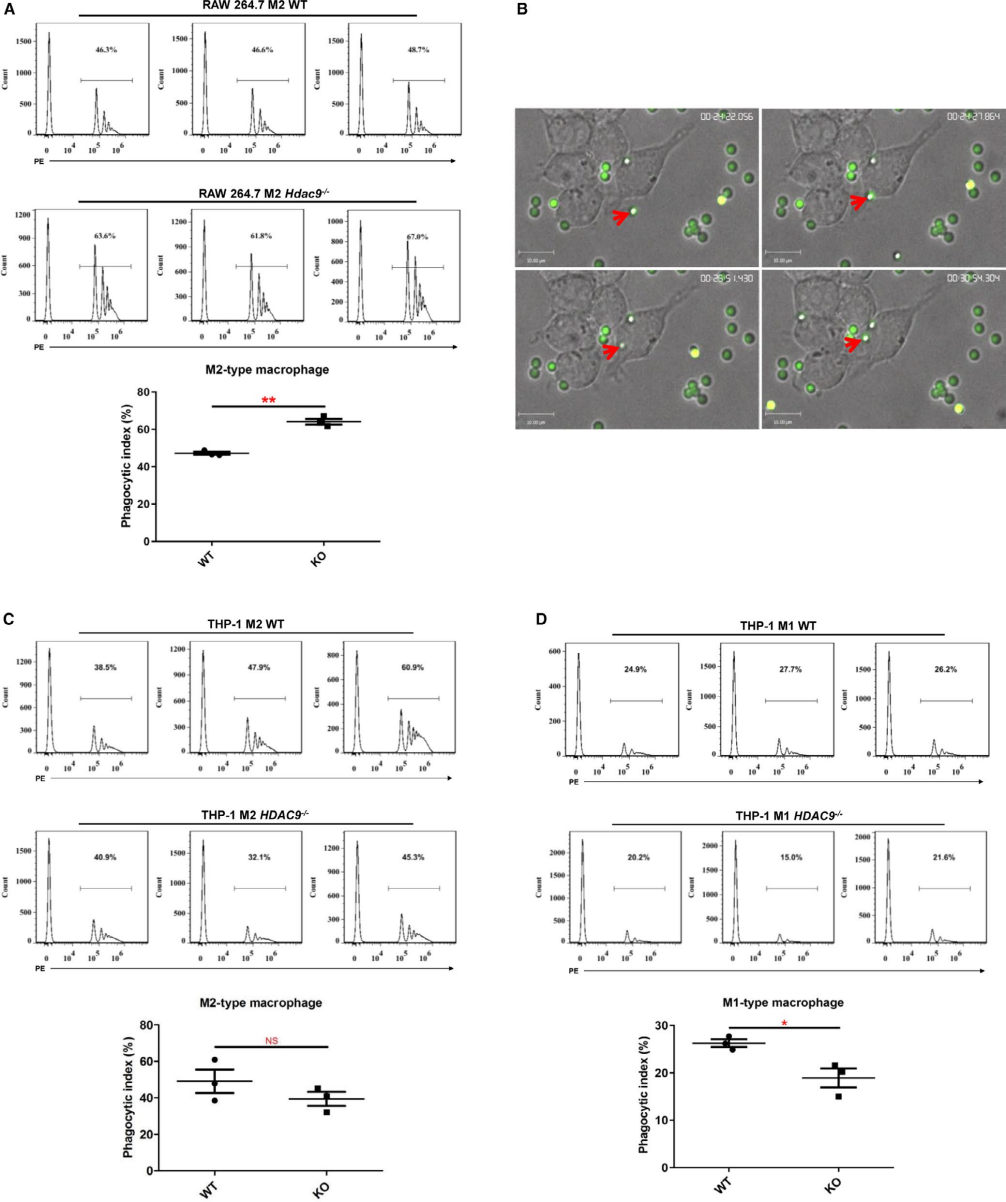
Effect of *Hdac9*/*HDAC9* deletion on the phagocytic capacity of RAW 264.7 and THP‐1‐derived macrophages by flow cytometry analysis. A, Phagocytosis assay of M2‐type RAW 264.7 cells. B, Real‐time live‐cell confocal recording of the process of polarized M2 RAW 264.7 cell phagocytosis of latex beads. C, Phagocytosis assay of M2‐type THP‐1‐derived macrophages. D, Phagocytosis assay of M1‐type THP‐1‐derived macrophages. The lower panels in A, C and D show the statistical summary of the percentage of phagocytic cells. **P < .01, *P < .05

Phagocytosis plays a crucial role in macrophage‐mediated host defence, which leads to internalization and distraction of pathogens. Raw 264.7 and THP‐1 cells have been demonstrated to be capable of phagocytosing red fluorescent latex beads of 2 μmol/L size by fluorescence measurements.^26^, ^27^, ^28^, ^29^ To determine whether *Hdac9* KO affected the phagocytosis of M2‐polarized macrophages, we examined the internalization of red fluorescent beads by FACS (Figure 7A,C,D) and real‐time live‐cell confocal imaging (Figure 7B, Supporting Video). The data showed that M2 cells after *Hdac9* deletion had higher capacity of uptaking latex beads in RAW 264.7 macrophages (Figure 7A). Although THP‐1‐derived M2 macrophages exhibited similar particle uptake rates between WT and KO cell lines (Figure 7C), *HDAC9* KO M1 macrophages showed decreased phagocytic capacity for latex beads (Figure 7D). These results further support the tendency towards M2 differentiation after *Hdac9*/*HDAC9* ablation.

## 4 DISCUSSION

F4/80, a well‐known surface marker of mouse macrophage, has also been known as a marker for discriminating or FACS sorting macrophages in mouse uterus.^10^, ^30^, ^31^ Two subsets of uterine Mϕs have been recognized, F4/80^+^MHCII^−^ and F4/80^+^MHCII^+^.^30^ The former subsets are defined as undifferentiated macrophages dependent on ovarian steroid hormones for maintenance, of which 70%‐80% express CD11b, but hardly express class A scavenger receptor, macrosialin or sialoadhesin. The latter subsets are defined as mature macrophages, half of which express CD11b, class A scavenger receptor, macrosialin and sialoadhesin.^30^ Additional studies also show that subpopulations of mouse dMϕs, defined by F4/80^+^MHCII^hi^ and F4/80^+^MHCII^lo^, differ in their dependence on CSF‐1R signalling for population accumulation and proliferation.^10^ Interestingly, MHCII^lo^ Mϕs in the E9.5‐10.5 myometrium express significantly higher mRNA levels of *Lyve1*, *Cd163*, *Stab1* (*stabilin‐1*) and *Cd206*, M2 phenotype markers associated with angiogenesis, tissue remodelling and repair, whereas MHCII^hi^ Mϕs show a trend towards higher expression of M1 markers.^10^ Although some studies have also proposed that F4/80 and CD11b are used together to FACS‐sort other tissue‐resident macrophages^32^ and bone marrow–derived monocytes/macrophages,^33^, ^34^ CD11b is more often referred as the monocyte marker, especially in the decidua.^35^ Based on these literatures, we used CD45 and F4/80 as the surface sorting marker to separate macrophages.

CD206 has been characterized as the marker of tissue M2 macrophages in both humans and mice, and M1 macrophages are devoid of it.^36^ In mice, aortic macrophage population has been divided into two distinct subpopulations, F4/80^+^CD206^−^iNOS^+^ (M1) and F4/80^+^CD206^+^iNOS^−^ (M2).^37^ Mouse M1 and M2 BMMs are defined as F4/80^+^CD11b^+^CD206^−^iNOS^+^ and F4/80^+^CD11b^+^CD206^+^iNOS^−^, respectively, after stimulation by polymer wear particles used in severe end‐stage arthritis.^34^ In addition, F4/80^+^CD11c^+^CD206^−^ M1 and F4/80^+^CD11c^−^CD206^+^ M2 cells reside in mouse epididymal fat tissue.^38^ Yet, in the uterus or the decidua, although it has been recognized that human dMϕs express more CD206 as compared with peripheral blood macrophages,^39^ there is no report on whether CD206 can be used as the surface sorting marker to discriminate M1 and M2. In this study, we firstly utilized antibodies against the surface epitope of CD206 and FACS‐sorted two distinct macrophage subpopulations, CD45^+^F4/80^+^CD206^−^ and CD45^+^F4/80^+^CD206^+^, and defined them as M1 and M2 cells, respectively. Indeed, F4/80^+^CD206^−^ (M1) and F4/80^+^CD206^+^ (M2) have also been characterized in mouse pancreas^40^ and infracted myocardium.^41^

It has been reported that adoptive transfer of Tregs,^42^, ^43^, ^44^ CD25^+^Foxp3^+^ NK cells^45^ and Tim‐3^+^ peripheral NK cells^46^ can protect against foetal loss in abortion‐prone mice. Adoptive transfer of RANK^+^ Mϕs, which are supposed to be more adaptive to M2 phenotype than RANK^−^ Mϕs, on gd5, reverses embryo absorption in pregnant C57BL/6 mice with macrophage depletion.^13^ Our data proved that LPS‐induced abortion was associated with the predominant M1 phenotype and that adoptive transfer of M2 cells partially rescued low‐dose LPS‐induced abortion in mice. As abortion is a shift in the immunological response from Th2 to Th1 domination, our results correlate with lines of evidence that adoptive transfer of M2 macrophages with immunosuppressive properties is an effective treatment of chronic pro‐inflammatory conditions in rodents.^47^, ^48^

As aforementioned, Hdacs mainly perform repressive function for gene transcription. Our results showed that *Hdac9* was highly expressed in M2 compared with M1 in in vivo isolated uterine and splenic macrophages, as well as in vitro PM, BMM and Raw 264.7 macrophages. Furthermore, *Hdac9*/*HDAC9* KO in Raw 264.7 and THP‐1 cells caused exaggerated M2 phenotype, including up‐regulation of M2 surface markers, decreased M1 marker gene expression and enhanced phagocytosis capacity. Likewise, genetic targeting or pharmacological inhibition of other *Hdac* members, such as *Hdac2*, *Hdac3*, *Hdac7* attenuates inflammatory responses and the expression of IL1b, IL6, IL12p40, TNF‐α, iNOS in LPS‐induced M1 macrophages,^16^, ^17^, ^49^, ^50^, ^51^ and triggers a more sensitive IL4‐induced M2 phenotype.^17^ In contrast, *Hdac6* KO BMMs show normal LPS‐induced expression of inflammatory genes *endothelin‐1* (*Edn‐1*) and *IL12p40*.^52^ Notably, Class IIa HDAC inhibitors attenuate inflammation in mouse and human macrophages,^49^, ^53^ stabilize atherosclerotic plaques in mice and limit the expression of inflammatory factors IL‐1β and IL‐6 in monocytes from patients with atherosclerosis, a chronic arterial inflammatory condition.^53^ Therefore, it is reasonable to consider that high‐level expression of HDAC9, a class IIa HDAC member, in M2 is involved in suppressing key genes that drive M2 differentiation.

*Hdac9* KO mice develop age‐dependent cardiac hypertrophy and heart failure,^54^ polydactyly,^55^ and are resistant to colitis,^56^ obesity and glucose intolerance during high‐fat feeding.^57^ *Hdac9*‐deficient mice also exhibit Th2 polarization in effector T cells via increased accumulation of H3, H3K9Ac, H3K14Ac and/or H3K18Ac at the promoters of *Il4*, *Roquin* and *Pparg* accompanied by increased expression of these genes in spleen and kidney.^58^ Besides phenotypes in T cells, several studies demonstrate the function of Hdac9 in macrophages. Compared with the single *LDLr* KO mice, *Hdac9* and *LDLr* double KO mice have increased cholesterol efflux and decreased atherosclerosis.^59^ Furthermore, *Hdac9* ^−/−^ *LDLr* ^−/−^ BMMs have increased expression of M2 markers and decreased expression of M1 inflammatory genes, resulting in macrophage polarization towards an M2‐like phenotype via increased total H3Ac, H4Ac and H3K9Ac at the promoters of *Pparg* and up‐regulated expression of *Pparg*.^59^ *Hdac9* ^–/–^ *Apoe* ^–/–^ BMMs have reduced TNF‐α‐induced up‐regulation of pro‐inflammatory gene expression, and during the development of atherosclerosis, HDAC9 binds to, deacetylates and activates inhibitory kappa B kinase (IKK)‐α and β, driving inflammatory responses in macrophages and endothelial cells.^53^ Interestingly, DNA methyltransferase Dnmt3a up‐regulates the expression of Hdac9, to deacetylate the key PRR signalling molecule TBK1 and to activate the transcription of type I interferon genes in primary PM during innate antiviral immunity.^60^ Taken together, these studies inspire us to suspect that Hdac9 plays conservative roles in uterine macrophages and other tissue‐resident macrophages.

To our knowledge, in uterine macrophages or in patients with gestational diseases, there are two studies addressing the function of HDAC members. HDAC8 expression is decreased in decidual macrophages from recurrent spontaneous miscarriage patients, and *HDAC8* knockdown suppresses M2 marker genes via activating ERK signalling pathway in THP‐1‐derived macrophages.^61^ HDAC2 is down‐regulated in the peripheral blood monocytes/macrophages from patients with gestational diabetes mellitus, which is characterized by high serum levels of pro‐inflammatory cytokines, and HDAC2 inhibition aggravates the secretion of pro‐inflammatory cytokines in the monocytes/macrophages.^62^ The effects of suppression of these two class I HDAC members on M1/M2 marker gene expression are not consistent with our results, suggesting distinct functions of different HDAC members in the local immunological environment at the maternal‐foetal interface. We speculate that HDAC9 mainly functions as an epigenetic brake in uterine M2 macrophages by suppressing the expression of M2 marker genes, for instance, CD206, CD209 and PPARG, and loss of HDAC9 thereby releases the brake and exaggerates M2 phenotype. It also remains possible that deacetylation of non‐histone proteins is mechanistically involved. In this perspective, our future studies will be aimed at defining the key HDAC9 target genes, including HDAC9‐marked enhancers/promotors and HDAC9 partners, during macrophage differentiation via high‐throughput omics approaches.

Accumulating evidence also reveals the function of HDAC9 in other inflammatory responses. HDAC9 deficiency alleviates the release of iNOS, cyclooxygenase‐2 (COX‐2), IL‐1β, IL‐6, TNF‐α and IL‐18 and suppresses inflammation in mouse brain via inactivating IkBa/NF‐kB and MAPKs signalling pathways.^63^ Prevented inflammation or decreased cytokine production by HDAC9 silencing or inhibition is also evident in splenocytes/kidneys due to PPARG overexpression,^58^ as well as in colitis via increasing Foxp3^+^ T regulatory cell function^56^ in mice. On the other hand, inflammation or LPS can drive the expression of HDAC9, triggering the activation of NF‐kB‐dependent inflammatory cytokines in microglial cells.^63^ HDAC9 is also up‐regulated after ischaemic brain injury which is associated with exacerbating inflammation in rats.^64^ Of note, genome‐wide association study identifies a variant in *HDAC9* associated with large vessel stroke, which would be consistent with the association with accelerating atherosclerosis.^65^ Overall, HDAC9 is an important epigenetic inflammatory mediator regulating the inflammatory gene expression programme, and targeting HDAC9 could be an effective strategy for ameliorating inflammation. Besides, pharmacologic HDAC inhibitors have considerable therapeutic benefits as anti‐inflammatory and immunosuppressive drugs in treating cancer, infectious and immunological diseases, etc^15^, ^66^ Based on available literatures, it is perplexing to understand the diverse HDAC functions. Our findings provide insights into Hdac9 as an epigenetic factor in maintaining immune homeostasis at the maternal‐foetal interface. It is therefore tempting to speculate the opportunities of using HDAC9 inhibitors as potential therapeutic strategies of inflammation‐related abortion.

## CONFLICT OF INTEREST

The authors confirm that there are no conflicts of interest.

## AUTHOR CONTRIBUTIONS

**Yanqin Liu:** Conceptualization (supporting); Data curation (lead); Formal analysis (lead); Investigation (lead); Methodology (equal); Resources (supporting); Validation (lead); Writing‐original draft (lead); Writing‐review & editing (lead). **Meirong Du:** Conceptualization (supporting); Data curation (supporting); Formal analysis (supporting); Investigation (supporting); Methodology (supporting); Resources (supporting); Validation (supporting); Writing‐review & editing (supporting). **Hai‐Yan Lin:** Conceptualization (lead); Data curation (lead); Formal analysis (lead); Funding acquisition (lead); Investigation (lead); Methodology (equal); Project administration (equal); Resources (equal); Supervision (lead); Validation (lead); Writing‐original draft (lead); Writing‐review & editing (lead).

## Supporting information

Fig S1

Fig S2

Supplementary Material

Table S1‐S6

Supplementary Material

## Data Availability

The data relevant to the study are included in the article. The data that support the findings of this study are available from the corresponding authors upon request.
