# Supplementary figures and images for: Histone deacetylase 9 deficiency exaggerates uterine M2 macrophage polarization
Source: J Cell Mol Med. 2021 Jun 19;25(16):7690–708. doi: 10.1111/jcmm.16616 (PMC8358884; doi:10.1111/jcmm.16616)

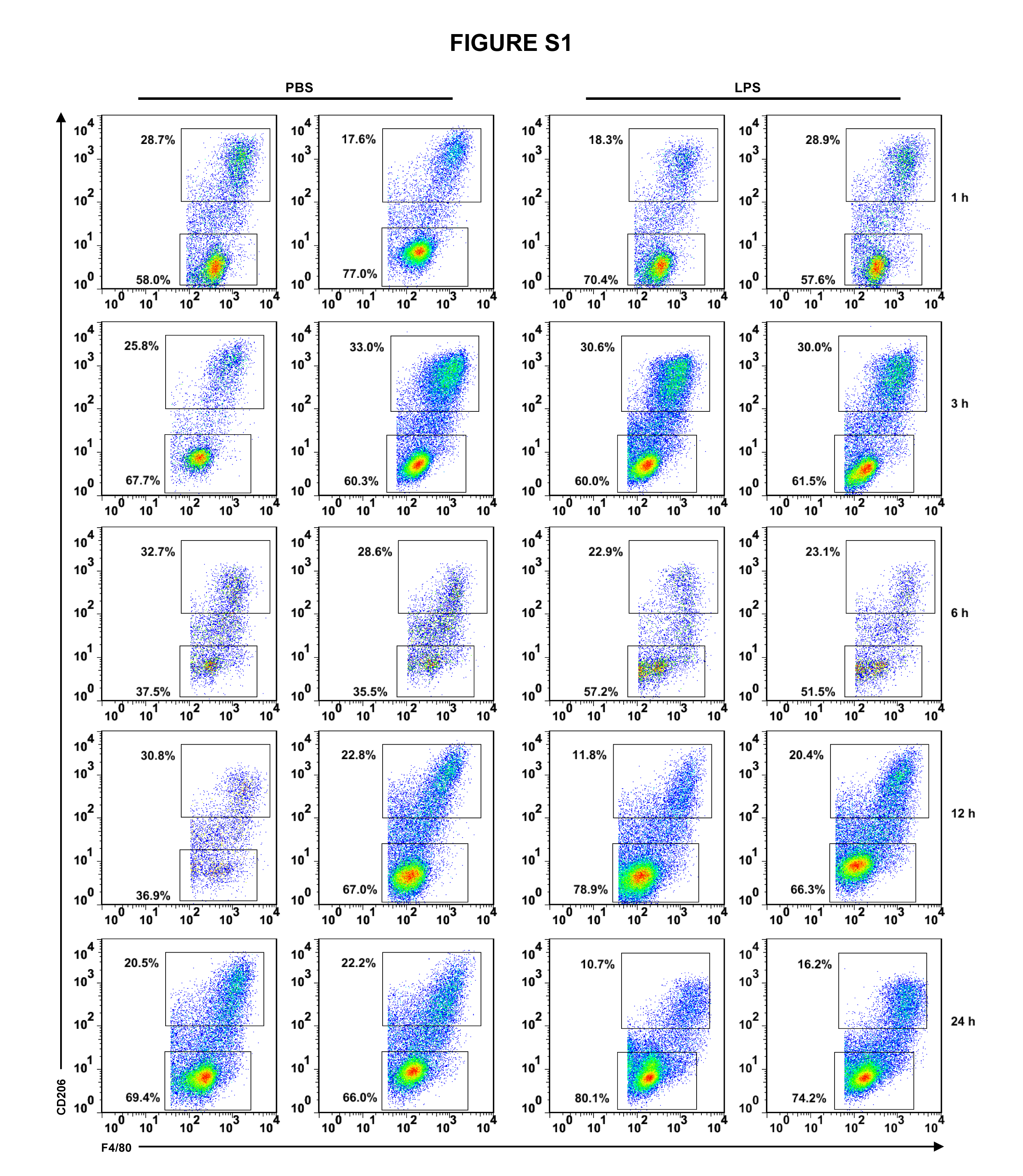

Supplement: Supplementary file 1 — Fig S1 [file JCMM-25-7690-s005.tif]

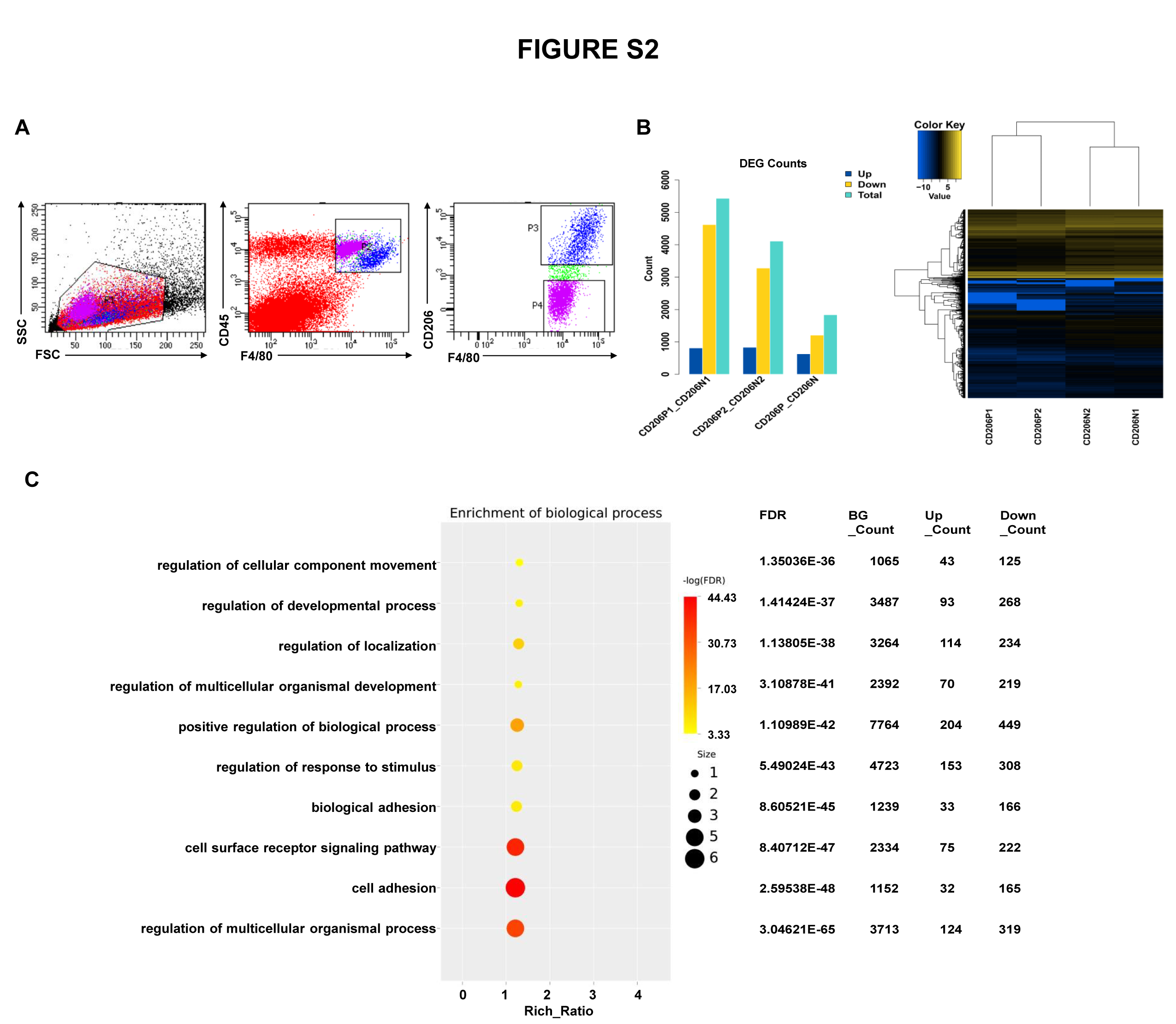

Supplement: Supplementary file 2 — Fig S2 [file JCMM-25-7690-s002.tif]
