# Supplementary material for: Histone deacetylase 9 deficiency exaggerates uterine M2 macrophage polarization
Source: J Cell Mol Med. 2021 Jun 19;25(16):7690–708. doi: 10.1111/jcmm.16616 (PMC8358884; doi:10.1111/jcmm.16616)
Supplement: Supplementary file 3 — Supplementary Material [file JCMM-25-7690-s001.docx]

**Supplementary Figure legends**

**FIGURE S1** Flow cytometric analysis of the proportions of M1- and M2-like macrophage subsets at 1, 3, 6, 12 and 24 h after LPS i.p. injection on gd6. The data show another two experimental replicates in Figure 1B.

**FIGURE S2** RNA-seq analysis of FACS-sorted mouse uterine M1-like and M2-like cells on gd6. (A) FACS gating strategy of M1- and M2-like cells. (B) The count and the heat map of the DEGs in the two uterine macrophage subsets. CD206P, M2; CD206N, M1. n = 2 biologically-independent samples. Up, upregulated genes in the former group; Down, downregulated genes in the former group; Total, sum of up- and down- regulated gene in the comparison group. (C) Top 10 enriched GO terms in the biological processes. FDR (false discovery rate), adjusted p-value; BG (background)_Count, count of all genes annotated in this GO Term; Up_Count: Count of upregulated genes annotated in this GO term; Down_Count: Count of downregulated genes annotated in this GO term.
