# Supplementary material for: Histone deacetylase 9 deficiency exaggerates uterine M2 macrophage polarization
Source: J Cell Mol Med. 2021 Jun 19;25(16):7690–708. doi: 10.1111/jcmm.16616 (PMC8358884; doi:10.1111/jcmm.16616)
Supplement: Supplementary file 4 — Table S1‐S6 [file JCMM-25-7690-s003.docx]

**Table S1. Primers used for qRT-PCR analysis**

| **Gene name**  **(Aliases)** | **NM Accession No.** | **Product length (bp)** | **Primer sequence (5'-3')** |
| --- | --- | --- | --- |
| mCd86 | NM_019388.3 | 100 | FF: TCTCCACGGAAACAGCATCT  R: CTTACGGAAGCACCCACGAT[^1^](#_ENREF_1) |
| mAdgre1  (F4/80, Emr1) | [NM_010130.4](https://www.ncbi.nlm.nih.gov/entrez/viewer.fcgi?db=nucleotide&id=183583543)  [NM_001355722.1](https://www.ncbi.nlm.nih.gov/entrez/viewer.fcgi?db=nucleotide&id=1246417732)  [NM_001355723.1](https://www.ncbi.nlm.nih.gov/entrez/viewer.fcgi?db=nucleotide&id=1246417652) | 166 | F: CTTTGGCTATGGGCTTCCAGTC  R: GGCAAGGAGGACAGAGTTTATCGTG[^2^](#_ENREF_2) |
| mNos2  (Inos) | [NM_010927.4](https://www.ncbi.nlm.nih.gov/entrez/viewer.fcgi?db=nucleotide&id=927028884)  [NM_001313921.1](https://www.ncbi.nlm.nih.gov/entrez/viewer.fcgi?db=nucleotide&id=927028885)  [NM_001313922.1](https://www.ncbi.nlm.nih.gov/entrez/viewer.fcgi?db=nucleotide&id=927028887) | 95 | F: CAGCTGGGCTGTACAAACCTT  R: CATTGGAAGTGAAGCGTTTCG[^3^](#_ENREF_3) |
| mCxcl10 | [NM_021274.2](https://www.ncbi.nlm.nih.gov/entrez/viewer.fcgi?db=nucleotide&id=371940989) | 157 | F: CCAAGTGCTGCCGTCATTTTC  R: GGCTCGCAGGGATGATTTCAA[^4^](#_ENREF_4) |
| mTnf  (Tnf-α) | [NM_013693.3](https://www.ncbi.nlm.nih.gov/entrez/viewer.fcgi?db=nucleotide&id=518831586)  [NM_001278601.1](https://www.ncbi.nlm.nih.gov/nuccore/NM_001278601.1) | 115 | F: GCCTCTTCTCATTCCTGCTTG  R: CTGATGAGAGGGAGGCCATT[^5^](#_ENREF_5) |
| mIl6 | [NM_031168.2](https://www.ncbi.nlm.nih.gov/entrez/viewer.fcgi?db=nucleotide&id=930945753)  [NM_001314054.1](https://www.ncbi.nlm.nih.gov/nuccore/NM_001314054.1) | 331 | F: TCCAGTTGCCTTCTTGGGAC  R: GTACTCCAGAAGACCAGAGG[^6^](#_ENREF_6) |
| mMrc1  (Cd206) | [NM_008625.2](https://www.ncbi.nlm.nih.gov/entrez/viewer.fcgi?db=nucleotide&id=224967061) | 118 | F: CAGGTGTGGGCTCAGGTAGT  R: TGTGGTGAGCTGAAAGGTGA[^1^](#_ENREF_1) |
| mArg1 | [NM_007482.3](https://www.ncbi.nlm.nih.gov/entrez/viewer.fcgi?db=nucleotide&id=158966684) | 140 | F: GGAATCTGCATGGGCAACCTGTGT  R: AGGGTCTACGTCTCGCAAGCCA[^7^](#_ENREF_7) |
| mPparg  (Nr1c3,  PPAR-gamma) | [NM_011146.3](https://www.ncbi.nlm.nih.gov/entrez/viewer.fcgi?db=nucleotide&id=187960102)  [NM_001127330.2](https://www.ncbi.nlm.nih.gov/nuccore/NM_001127330.2)  [NM_001308352.1](https://www.ncbi.nlm.nih.gov/nuccore/NM_001308352.1) | 120 | F: TGTGGGGATAAAGCATCAGGC  R: CCGGCAGTTAAGATCACACCTAT[^8^](#_ENREF_8) |
| mCd163 |  |  | F: GCAAAAACTGGCAGTGGG  R:GTCAAAATCACAGACGGAGC |
| mGapdh | [NM_001289726.1](https://www.ncbi.nlm.nih.gov/entrez/viewer.fcgi?db=nucleotide&id=576080554)  [NM_008084.3](https://www.ncbi.nlm.nih.gov/nuccore/NM_008084.3) | 123 | F: AGGTCGGTGTGAACGGATTTG  R: TGTAGACCATGTAGTTGAGGTCA[^9^](#_ENREF_9) |
| mHdac7 | [NM_001204275.1](https://www.ncbi.nlm.nih.gov/entrez/viewer.fcgi?db=nucleotide&id=324073113)  [NM_001204280.1](https://www.ncbi.nlm.nih.gov/entrez/viewer.fcgi?db=nucleotide&id=324073185)  [NM_001204281.1](https://www.ncbi.nlm.nih.gov/entrez/viewer.fcgi?db=nucleotide&id=324073183)  [NM_001204279.1](https://www.ncbi.nlm.nih.gov/entrez/viewer.fcgi?db=nucleotide&id=324073169)  [NM_001204278.1](https://www.ncbi.nlm.nih.gov/entrez/viewer.fcgi?db=nucleotide&id=324073149)  [NM_001204277.1](https://www.ncbi.nlm.nih.gov/entrez/viewer.fcgi?db=nucleotide&id=324073137)  [NM_001204276.1](https://www.ncbi.nlm.nih.gov/entrez/viewer.fcgi?db=nucleotide&id=324073134)  [NM_019572.3](https://www.ncbi.nlm.nih.gov/entrez/viewer.fcgi?db=nucleotide&id=324073098) | 218 | F: TGGTCAGGGTGCACAGGAAAT  R: AGCGCGATCTGTTCTGAAACC |
| mHdac8 | [NM_027382.4](https://www.ncbi.nlm.nih.gov/entrez/viewer.fcgi?db=nucleotide&id=924183166)  [NM_001313742.1](https://www.ncbi.nlm.nih.gov/entrez/viewer.fcgi?db=nucleotide&id=924182598) | 150 | F: GTCAGCCAAGAAGGTGATGAGG  R: ACACTTCCCGTCAATCAGGCAC |
| mHdac9 | [NM_001271386.1](https://www.ncbi.nlm.nih.gov/entrez/viewer.fcgi?db=nucleotide&id=405978896)  [NM_024124.3](https://www.ncbi.nlm.nih.gov/entrez/viewer.fcgi?db=nucleotide&id=237649054) | 214 | F: CGACCCCTGAATCGAACCCA  R: TGGATTGGTCCCCCTGAAGC |
| mHdac9-q-  ex2F-ex3R | [NM_001271386.1](https://www.ncbi.nlm.nih.gov/entrez/viewer.fcgi?db=nucleotide&id=405978896)  [NM_024124.3](https://www.ncbi.nlm.nih.gov/entrez/viewer.fcgi?db=nucleotide&id=237649054) | 132 | F: TGGCTCAGCAAAGAATGCACAG  R: CAGGATCCACCACAGGCATCA |
| hCD68 | [NM_001251.2](https://www.ncbi.nlm.nih.gov/entrez/viewer.fcgi?db=nucleotide&id=91199547)  [NM_001040059.1](https://www.ncbi.nlm.nih.gov/nuccore/NM_001040059.1) | 202 | F: TCAGCTTTGGATTCATGCAG  R: AGGTGGACAGCTGGTGAAAG[^10^](#_ENREF_10) |
| hCD14 | [NM_000591.3](https://www.ncbi.nlm.nih.gov/entrez/viewer.fcgi?db=nucleotide&id=291575160)  [NM_001040021.2](https://www.ncbi.nlm.nih.gov/nuccore/NM_001040021.2)  [NM_001174104.1](https://www.ncbi.nlm.nih.gov/nuccore/NM_001174104.1)  [NM_001174105.1](https://www.ncbi.nlm.nih.gov/nuccore/NM_001174105.1) | 122 | F: ACGCCAGAACCTTGTGAGC  R: GCATGGATCTCCACCTCTACTG[^11^](#_ENREF_11) |
| hTNF  (TNF-α) | [NM_000594.3](https://www.ncbi.nlm.nih.gov/entrez/viewer.fcgi?db=nucleotide&id=395132451) | 217 | F: ATGAGCACTGAAAGCATGATCC  R: GAGGGCTGATTAGAGAGAGGTC[^12^](#_ENREF_12) |
| hIL6 | [NM_000600.4](https://www.ncbi.nlm.nih.gov/entrez/viewer.fcgi?db=nucleotide&id=969812508)  [NM_001318095.1](https://www.ncbi.nlm.nih.gov/nuccore/NM_001318095.1) | 101 | F: AATTCGGTACATCCTCGACGG  R: GGTTGTTTTCTGCCAGTGCC[^13^](#_ENREF_13) |
| hCXCL10 | [NM_001565.3](https://www.ncbi.nlm.nih.gov/entrez/viewer.fcgi?db=nucleotide&id=323422857) | 193 | F: GTGGCATTCAAGGAGTACCTC  R: GCCTTCGATTCTGGATTCAG[^14^](#_ENREF_14) |
| hMRC1  (CD206) | [NM_002438.3](https://www.ncbi.nlm.nih.gov/entrez/viewer.fcgi?db=nucleotide&id=471270251) | 84 | F: CGAGGAAGAGGTTCGGTTCACC  R: GCAATCCCGGTTCTCATGGC[^15^](#_ENREF_15) |
| hCD209  (DC-SIGN) | [NM_021155.3](https://www.ncbi.nlm.nih.gov/entrez/viewer.fcgi?db=nucleotide&id=223029378)  [NM_001144899.1](https://www.ncbi.nlm.nih.gov/entrez/viewer.fcgi?db=nucleotide&id=223029516)  [NM_001144897.1](https://www.ncbi.nlm.nih.gov/entrez/viewer.fcgi?db=nucleotide&id=223029387)  [NM_001144896.1](https://www.ncbi.nlm.nih.gov/entrez/viewer.fcgi?db=nucleotide&id=223029385)  [NM_001144895.1](https://www.ncbi.nlm.nih.gov/entrez/viewer.fcgi?db=nucleotide&id=223029383)  [NM_001144894.1](https://www.ncbi.nlm.nih.gov/entrez/viewer.fcgi?db=nucleotide&id=223029381)  [NM_001144893.1](https://www.ncbi.nlm.nih.gov/entrez/viewer.fcgi?db=nucleotide&id=223029379) | 149 | F: CCTGGATGGGACTTTCAGATC  R: GCCATTGCCACTAAATTCCG[^16^](#_ENREF_16) |
| hGAPDH | [NM_002046.6](https://www.ncbi.nlm.nih.gov/entrez/viewer.fcgi?db=nucleotide&id=1276346088)  [NM_001289746.1](https://www.ncbi.nlm.nih.gov/entrez/viewer.fcgi?db=nucleotide&id=576583523)  [NM_001289745.2](https://www.ncbi.nlm.nih.gov/entrez/viewer.fcgi?db=nucleotide&id=1276346089)  [NM_001357943.1](https://www.ncbi.nlm.nih.gov/entrez/viewer.fcgi?db=nucleotide&id=1276317823) | 315 | F: AGCCACATCGCTCAGACA  R: TGGACTCCACGACGTACT[^17^](#_ENREF_17) |
| hHDAC9 | [NM_058176.2](https://www.ncbi.nlm.nih.gov/entrez/viewer.fcgi?db=nucleotide&id=116284378)  [NM_001321902.1](https://www.ncbi.nlm.nih.gov/entrez/viewer.fcgi?db=nucleotide&id=1013403381)  [NM_001321901.1](https://www.ncbi.nlm.nih.gov/entrez/viewer.fcgi?db=nucleotide&id=1013403379)  [NM_001321900.1](https://www.ncbi.nlm.nih.gov/entrez/viewer.fcgi?db=nucleotide&id=1013403373)  [NM_001321899.1](https://www.ncbi.nlm.nih.gov/entrez/viewer.fcgi?db=nucleotide&id=1013403348)  [NM_001321898.1](https://www.ncbi.nlm.nih.gov/entrez/viewer.fcgi?db=nucleotide&id=1013403323)  [NM_001321897.1](https://www.ncbi.nlm.nih.gov/entrez/viewer.fcgi?db=nucleotide&id=1013403283)  [NM_001321896.1](https://www.ncbi.nlm.nih.gov/entrez/viewer.fcgi?db=nucleotide&id=1013403261)  [NM_001321895.1](https://www.ncbi.nlm.nih.gov/entrez/viewer.fcgi?db=nucleotide&id=1013403216)  [NM_001321894.1](https://www.ncbi.nlm.nih.gov/entrez/viewer.fcgi?db=nucleotide&id=1013403176)  [NM_001321891.1](https://www.ncbi.nlm.nih.gov/entrez/viewer.fcgi?db=nucleotide&id=1013403136)  [NM_001321893.1](https://www.ncbi.nlm.nih.gov/entrez/viewer.fcgi?db=nucleotide&id=1013403131)  [NM_001321890.1](https://www.ncbi.nlm.nih.gov/entrez/viewer.fcgi?db=nucleotide&id=1013403058)  [NM_001321889.1](https://www.ncbi.nlm.nih.gov/entrez/viewer.fcgi?db=nucleotide&id=1013403029)  [NM_001321888.1](https://www.ncbi.nlm.nih.gov/entrez/viewer.fcgi?db=nucleotide&id=1013403000)  [NM_001321887.1](https://www.ncbi.nlm.nih.gov/entrez/viewer.fcgi?db=nucleotide&id=1013402973)  [NM_001321886.1](https://www.ncbi.nlm.nih.gov/entrez/viewer.fcgi?db=nucleotide&id=1013402945)  [NM_001321885.1](https://www.ncbi.nlm.nih.gov/entrez/viewer.fcgi?db=nucleotide&id=1013402924)  [NM_001321884.1](https://www.ncbi.nlm.nih.gov/entrez/viewer.fcgi?db=nucleotide&id=1013402883)  [NM_001321879.1](https://www.ncbi.nlm.nih.gov/entrez/viewer.fcgi?db=nucleotide&id=1013402846)  [NM_001321878.1](https://www.ncbi.nlm.nih.gov/entrez/viewer.fcgi?db=nucleotide&id=1013402801)  [NM_001321877.1](https://www.ncbi.nlm.nih.gov/entrez/viewer.fcgi?db=nucleotide&id=1013402770)  [NM_001321876.1](https://www.ncbi.nlm.nih.gov/entrez/viewer.fcgi?db=nucleotide&id=1013402759)  [NM_001321875.1](https://www.ncbi.nlm.nih.gov/entrez/viewer.fcgi?db=nucleotide&id=1013402723)  [NM_001321874.1](https://www.ncbi.nlm.nih.gov/entrez/viewer.fcgi?db=nucleotide&id=1013402696)  [NM_001321873.1](https://www.ncbi.nlm.nih.gov/entrez/viewer.fcgi?db=nucleotide&id=1013402655)  [NM_001321872.1](https://www.ncbi.nlm.nih.gov/entrez/viewer.fcgi?db=nucleotide&id=1013402609)  [NM_001321871.1](https://www.ncbi.nlm.nih.gov/entrez/viewer.fcgi?db=nucleotide&id=1013402578)  [NM_001321870.1](https://www.ncbi.nlm.nih.gov/entrez/viewer.fcgi?db=nucleotide&id=1013402568)  [NM_001321869.1](https://www.ncbi.nlm.nih.gov/entrez/viewer.fcgi?db=nucleotide&id=1013402566)  [NM_001321868.1](https://www.ncbi.nlm.nih.gov/entrez/viewer.fcgi?db=nucleotide&id=1013402294)  [NM_001204148.2](https://www.ncbi.nlm.nih.gov/entrez/viewer.fcgi?db=nucleotide&id=1013401534)  [NM_001204147.2](https://www.ncbi.nlm.nih.gov/entrez/viewer.fcgi?db=nucleotide&id=1013401198)  [NM_178425.3](https://www.ncbi.nlm.nih.gov/entrez/viewer.fcgi?db=nucleotide&id=1013400628)  [NM_001204145.2](https://www.ncbi.nlm.nih.gov/entrez/viewer.fcgi?db=nucleotide&id=1013400129)  [NM_001204146.2](https://www.ncbi.nlm.nih.gov/entrez/viewer.fcgi?db=nucleotide&id=1013400127)  [NM_014707.3](https://www.ncbi.nlm.nih.gov/entrez/viewer.fcgi?db=nucleotide&id=1013399451)  [NM_178423.2](https://www.ncbi.nlm.nih.gov/entrez/viewer.fcgi?db=nucleotide&id=1013398814)  [NM_001204144.2](https://www.ncbi.nlm.nih.gov/entrez/viewer.fcgi?db=nucleotide&id=1013398813)  [NM_001321884.1](https://www.ncbi.nlm.nih.gov/entrez/viewer.fcgi?db=nucleotide&id=1013402883) | 207 | F: GGTTTTGCCCCCTACCCCTC  R: CCTTGCCTAAGCGTCTGCGT |

**Table S2. Primers used for CRISPR-Cas9-mediated Hdac9 ablation**

| **Primer name** | **Primer sequence (5'-3')** |
| --- | --- |
| mHdac9 CRISPR gRNA oligo upstream pair 1 | F: CACCGTGTGCTTACATGCGCTATTC  R: AAACGAATAGCGCATGTAAGCACAC |
| mHdac9 CRISPR gRNA oligo upstream pair 2 | F: CACCGAGCTTTGGCAAAGGGTACAT  R: AAACATGTACCCTTTGCCAAAGCTC |
| mHdac9 CRISPR gRNA oligo downstream pair 3 | F: CACCGTGGGCATAAGACCGACCCA  R: AAACTGGGTCGGTCTTATGCCCAC |
| mHdac9 CRISPR gRNA oligo downstream pair 4 | F: CACCGTCGGTCTTATGCCCACATCA  R: AAACTGATGTGGGCATAAGACCGAC |
| mHdac9 knockout genotyping oligo pair (PCR product 965 bp) | F: TCTCCCATGTCCTTCCCAGGTGTAAT  R: ATCAGCAGAGCTCTCCCACTACCAA |
| hHdac9 CRISPR gRNA oligo upstream pair 1 | F: CACCGAAGGTCTTAACGTCATCAAC  R: AAACGTTGATGACGTTAAGACCTTC |
| hHdac9 CRISPR gRNA oligo downstream pair 3 | F: CACCGTCTAACCGCACTCTTTATC  R: AAACGATAAAGAGTGCGGTTAGAC |
| hHdac9 CRISPR gRNA oligo downstream pair 4 | F: CACCGATAATGATCAGCTAACGATG  R: AAACCATCGTTAGCTGATCATTATC |
| hHdac9 knockout genotyping oligo pair (PCR product 993 bp) | F: TCTCACCATTCTGTATTTGCGTTGA  R: AATGGAGGCAAGGCTTTACTTAGAG |

**Table S3. Number of reads and mapping efficiency of each sample**

| **Sample** | **CD206N1** | **CD206N2** | **CD206P1** | **CD206P2** |
| --- | --- | --- | --- | --- |
| Total reads number | 45,947,744 | 46,191,072 | 47,948,330 | 47,372,222 |
| Mapped reads number | 41,229,978 | 43,826,822 | 43,705,966 | 45,325,129 |
| Mapping rate (%) | 89.73 | 94.88 | 91.15 | 95.68 |

**Table S4. Top 10 enriched GO terms in molecular functions**

| **GO term** | **FDR** | **Background_Count** | **Up_Count** | **Down_Count** |
| --- | --- | --- | --- | --- |
| Protein binding | 3.97E-17 | 8983 | 194 | 436 |
| Carbohydrate binding | 5.27E-16 | 361 | 25 | 40 |
| Receptor binding | 5.27E-16 | 1931 | 59 | 132 |
| Cytokine receptor activity | 8.44E-11 | 96 | 8 | 19 |
| Transmembrane transporter activity | 8.44E-11 | 1375 | 55 | 80 |
| Inorganic molecular entity transmembrane transporter activity | 8.44E-11 | 1017 | 47 | 62 |
| Glycosaminoglycan binding | 2.87E-10 | 347 | 15 | 38 |
| Calcium ion binding | 3.47E-10 | 1059 | 19 | 91 |
| Ion transmembrane transporter activity | 6.2E-10 | 1112 | 49 | 64 |
| Cargo receptor activity | 3.1E-09 | 76 | 6 | 16 |

FDR (false discovery rate), adjusted p-value using multiple comparisons fallacy; Background_Count, count of all genes annotated in this GO Term; Up_Count: Count of up-regulated genes annotated in this GO term; Down_Count: Count of down-regulated genes annotated in this GO term.

**Table S5. Top 10 enriched GO terms in cellular components**

| **GO term** | **FDR** | **Background_Count** | **Up_Count** | **Down_Count** |
| --- | --- | --- | --- | --- |
| Plasma membrane part | 5.05E-94 | 3106 | 132 | 315 |
| Membrane part | 1.77E-68 | 10615 | 298 | 593 |
| Intrinsic component of membrane | 1.78E-53 | 8422 | 239 | 483 |
| Integral component of membrane | 3.47E-47 | 8213 | 233 | 458 |
| Intrinsic component of plasma membrane | 9.94E-43 | 1365 | 67 | 138 |
| Integral component of plasma membrane | 6.21E-39 | 1276 | 61 | 129 |
| Extracellular region part | 2.6E-38 | 6599 | 177 | 388 |
| Cell surface | 5.39E-32 | 775 | 30 | 101 |
| External side of plasma membrane | 5.28E-31 | 403 | 29 | 61 |
| Plasma membrane region | 6.2E-30 | 1202 | 42 | 124 |

FDR, adjusted p-value using multiple comparisons fallacy; Background_Count, count of all genes annotated in this GO Term; Up_Count: Count of up-regulated genes annotated in this GO term; Down_Count: Count of down-regulated genes annotated in this GO term.

**Table S6. KEGG pathway enrichment of DEGs**

| **Pathway** | **q Value** | **Up_**  **Count^†^** | **Down_**  **Count^†^** |
| --- | --- | --- | --- |
| Cytokine-cytokine receptor interaction | 1.25E-14 | 19 | 34 |
| Cell adhesion molecules (CAMs) | 1.62E-14 | 11 | 30 |
| Hematopoietic cell lineage | 1.81E-11 | 11 | 17 |
| Complement and coagulation cascades | 3.18E-11 | 10 | 18 |
| ECM-receptor interaction | 3.18E-11 | 2 | 24 |
| Axon guidance | 1.37E-09 | 9 | 31 |
| Lysosome | 9.37E-08 | 26 | 4 |
| Staphylococcus aureus infection | 8.15E-07 | 12 | 4 |
| Malaria | 9.58E-07 | 6 | 10 |
| Th1 and Th2 cell differentiation | 1.14E-06 | 5 | 16 |
| Pathways in cancer | 6.91E-06 | 17 | 61 |
| Th17 cell differentiation | 9.04E-06 | 8 | 16 |
| Basal cell carcinoma | 1.45E-05 | 2 | 14 |
| Focal adhesion | 1.45E-05 | 5 | 32 |
| Calcium signaling pathway | 3.16E-05 | 11 | 20 |
| Rheumatoid arthritis | 3.16E-05 | 11 | 8 |
| Chagas disease (American trypanosomiasis) | 3.23E-05 | 10 | 11 |
| cAMP signaling pathway | 7.97E-05 | 10 | 22 |
| PI3K-Akt signaling pathway | 7.97E-05 | 8 | 49 |
| Rap1 signaling pathway | 0.000187 | 5 | 31 |
| Proximal tubule bicarbonate reclamation | 0.000269 | 2 | 7 |
| Neuroactive ligand-receptor interaction | 0.000277 | 10 | 26 |
| Primary immunodeficiency | 0.000382 | 5 | 5 |
| Renin secretion | 0.000382 | 4 | 11 |
| MAPK signaling pathway | 0.00045 | 13 | 27 |
| Arrhythmogenic right ventricular cardiomyopathy (ARVC) | 0.000451 | 2 | 15 |
| Mineral absorption | 0.000451 | 3 | 10 |
| AGE-RAGE signaling pathway in diabetic complications | 0.000467 | 4 | 16 |
| Endocrine and other factor-regulated calcium reabsorption | 0.000467 | 7 | 7 |
| Transcriptional misregulation in cancer | 0.000695 | 14 | 17 |
| Human papillomavirus infection | 0.000852 | 5 | 39 |
| Salivary secretion | 0.000871 | 6 | 9 |
| Bile secretion | 0.000932 | 6 | 9 |
| Pertussis | 0.000932 | 11 | 6 |
| Wnt signaling pathway | 0.001109 | 4 | 20 |
| Melanogenesis | 0.001181 | 3 | 14 |
| Toll-like receptor signaling pathway | 0.001181 | 13 | 4 |
| Jak-STAT signaling pathway | 0.001317 | 8 | 15 |
| Synaptic vesicle cycle | 0.001368 | 11 | 3 |
| Thyroid hormone synthesis | 0.001368 | 4 | 10 |
| ABC transporters | 0.002231 | 5 | 6 |
| Inflammatory bowel disease (IBD) | 0.002231 | 7 | 4 |
| Leukocyte transendothelial migration | 0.003028 | 2 | 20 |
| Dilated cardiomyopathy (DCM) | 0.00396 | 5 | 12 |
| cGMP-PKG signaling pathway | 0.004779 | 6 | 19 |
| Cholesterol metabolism | 0.004779 | 9 | 2 |
| Relaxin signaling pathway | 0.006025 | 4 | 15 |
| Tuberculosis | 0.006226 | 17 | 8 |
| Hypertrophic cardiomyopathy (HCM) | 0.006498 | 3 | 13 |
| Natural killer cell mediated cytotoxicity | 0.006944 | 3 | 13 |
| Chemokine signaling pathway | 0.007014 | 16 | 9 |
| Gastric cancer | 0.008372 | 2 | 18 |
| NF-kappa B signaling pathway | 0.008372 | 8 | 8 |
| Phospholipase D signaling pathway | 0.008372 | 6 | 15 |
| Osteoclast differentiation | 0.010434 | 12 | 6 |
| Gastric acid secretion | 0.011418 | 4 | 8 |
| Hedgehog signaling pathway | 0.011747 | 2 | 7 |
| Epithelial cell signaling in Helicobacter pylori infection | 0.012186 | 5 | 7 |
| Glycosphingolipid biosynthesis - globo and isoglobo series | 0.015882 | 2 | 2 |
| Adrenergic signaling in cardiomyocytes | 0.015984 | 8 | 12 |
| Fluid shear stress and atherosclerosis | 0.015984 | 8 | 19 |
| Regulation of lipolysis in adipocytes | 0.016271 | 3 | 7 |
| Endocytosis | 0.01829 | 21 | 17 |
| Ras signaling pathway | 0.01829 | 7 | 22 |
| Regulation of actin cytoskeleton | 0.01829 | 8 | 20 |
| Tight junction | 0.01829 | 5 | 24 |
| Protein digestion and absorption | 0.018564 | 2 | 11 |
| Choline metabolism in cancer | 0.020345 | 4 | 12 |
| Amoebiasis | 0.024527 | 5 | 11 |
| Glycosaminoglycan degradation | 0.02468 | 5 | 0 |
| Thyroid cancer | 0.02468 | 3 | 6 |
| Phagosome | 0.025324 | 18 | 7 |
| Sphingolipid metabolism | 0.026671 | 6 | 2 |
| Glutamatergic synapse | 0.031491 | 7 | 9 |
| Breast cancer | 0.031993 | 2 | 18 |
| Adherens junction | 0.035315 | 2 | 13 |
| Amino sugar and nucleotide sugar metabolism | 0.041731 | 9 | 0 |

**^†^**Up_Count: Count of up-regulated genes in the KEGG pathway; Down_Count: Count of down-regulated genes in the KEGG pathway.

**REFERENCES**

1. Shaul ME, Bennett G, Strissel KJ, Greenberg AS, Obin MS. Dynamic, M2-like remodeling phenotypes of CD11c+ adipose tissue macrophages during high-fat diet--induced obesity in mice. *Diabetes*. 2010;59:1171-1181.

2. Li L, Ng DS, Mah WC, et al. A unique role for p53 in the regulation of M2 macrophage polarization. *Cell Death Differ*. 2015;22:1081-1093.

3. Alnaeeli M, Raaka BM, Gavrilova O, Teng R, Chanturiya T, Noguchi CT. Erythropoietin signaling: a novel regulator of white adipose tissue inflammation during diet-induced obesity. *Diabetes*. 2014;63:2415-2431.

4. Zheng QY, Liang SJ, Li GQ, et al. Complement component 3 deficiency prolongs MHC-II disparate skin allograft survival by increasing the CD4(+) CD25(+) regulatory T cells population. *Sci Rep*. 2016;6:33489.

5. Yamakawa I, Kojima H, Terashima T, et al. Inactivation of TNF-alpha ameliorates diabetic neuropathy in mice. *Am J Physiol Endocrinol Metab*. 2011;301:E844-852.

6. Kawane K, Tanaka H, Kitahara Y, Shimaoka S, Nagata S. Cytokine-dependent but acquired immunity-independent arthritis caused by DNA escaped from degradation. *Proc Natl Acad Sci U S A*. 2010;107:19432-19437.

7. Duque-Correa MA, Kuhl AA, Rodriguez PC, et al. Macrophage arginase-1 controls bacterial growth and pathology in hypoxic tuberculosis granulomas. *Proc Natl Acad Sci U S A*. 2014;111:E4024-4032.

8. Tan CK, Leuenberger N, Tan MJ, et al. Smad3 deficiency in mice protects against insulin resistance and obesity induced by a high-fat diet. *Diabetes*. 2011;60:464-476.

9. Xin Q, Li J, Dang J, et al. miR-155 Deficiency Ameliorates Autoimmune Inflammation of Systemic Lupus Erythematosus by Targeting S1pr1 in Faslpr/lpr Mice. *J Immunol*. 2015;194:5437-5445.

10. Young HL, Rowling EJ, Bugatti M, et al. An adaptive signaling network in melanoma inflammatory niches confers tolerance to MAPK signaling inhibition. *J Exp Med*. 2017;214:1691-1710.

11. Liu J, Ibi D, Taniguchi K, et al. Inflammation Improves Glucose Homeostasis through IKKbeta-XBP1s Interaction. *Cell*. 2016;167:1052-1066 e1018.

12. Kinoshita T, Imamura R, Kushiyama H, Suda T. NLRP3 mediates NF-kappaB activation and cytokine induction in microbially induced and sterile inflammation. *PLoS One*. 2015;10:e0119179.

13. Pfaffl MW. A new mathematical model for relative quantification in real-time RT-PCR. *Nucleic Acids Res*. 2001;29:e45.

14. Marroqui L, Dos Santos RS, Floyel T, et al. TYK2, a Candidate Gene for Type 1 Diabetes, Modulates Apoptosis and the Innate Immune Response in Human Pancreatic beta-Cells. *Diabetes*. 2015;64:3808-3817.

15. El Hadri K, Mahmood DF, Couchie D, et al. Thioredoxin-1 promotes anti-inflammatory macrophages of the M2 phenotype and antagonizes atherosclerosis. *Arterioscler Thromb Vasc Biol*. 2012;32:1445-1452.

16. Ali MF, Driscoll CB, Walters PR, Limper AH, Carmona EM. beta-Glucan-Activated Human B Lymphocytes Participate in Innate Immune Responses by Releasing Proinflammatory Cytokines and Stimulating Neutrophil Chemotaxis. *J Immunol*. 2015;195:5318-5326.

17. Tang F, Tang S, Guo X, Yang C, Jia K. CT45A1 siRNA silencing suppresses the proliferation, metastasis and invasion of lung cancer cells by downregulating the ERK/CREB signaling pathway. *Mol Med Rep*. 2017;16:6708-6714.
